# Study on magnetohydrodynamic internal cooling mechanism within an aluminium oxide cutting tool

**DOI:** 10.1007/s00170-024-13542-7

**Published:** 2024-04-22

**Authors:** John O’Hara, Fengzhou Fang

**Affiliations:** 1 Center of Micro/Nano Manufacturing Technology (MNMT-Dublin), University College Dublin, Dublin 4, Ireland; 2 State Key Laboratory of Precision Measuring Technology and Instruments, Laboratory of Micro/Nano Manufacturing Technology (MNMT), Tianjin University, Tianjin, 300072 China

**Keywords:** Heat transfer, Internal cooling, Magnetohydrodynamic drive, Liquid metal, Tool wear

## Abstract

One of the challenges in the transfer of heat during the mechanical machining process is the coolant substance used in the internal cooling method which is generally liquid water or a water-based coolant. This limits the heat transfer capacity insofar as the thermal conductivity of liquid water is concerned. The other difficulty is the requirement for an external mechanical system to pump the coolant around the internal channel, providing efficient transfer of the accumulated thermal energy. This study proposes a novel method to address this issue by using liquid gallium which provides the means to transfer the excess heat generated during the cutting process by integrating the design into an aluminium oxide insert. Combining this with a magnetohydrodynamic drive, the coolant system operates without the need for mechanical input. Liquid gallium is nontoxic and has a much higher thermal conductivity over liquid water. Investigations of the novel cooling system is performance compared against liquid water through numerical modelling, followed by an experimental machining test to ascertain the difference in heat transfer effectiveness, tool wear rates and workpiece surface finish when compared to dry machining and external cooling conditions on stainless steel 316L. Without cooling, experimental machining tests employing a cutting speed of V_c_ = 250 m min^−1^ resulted in a corner wear VB_c_ rate of 75 μm, and with the magnetohydrodynamic-based coolant on, produced a VB_c_ rate of 48 μm, indicating a difference of 36% in relative tool wear under the same cutting conditions. Increasing the cutting speed V_c_ to 900 m min^−1^, produced a corner wear VB_c_ rate of 357 μm without the active coolant and a VB_c_ rate of 246 μm with the magnetohydrodynamic-based coolant on, representing a decrease of 31% in relative tool wear. Further tests comparing external liquid water cooling against the liquid gallium coolant showed at V_c_ = 250 m min^−1^, a difference of 29% in relative tool wear rate reduction was obtained with the internal liquid gallium coolant. Increasing the cutting speed to V_c_ = 900 m min^−1^, the data indicated a difference of 16% relative tool wear reduction with the internal liquid gallium. The results support the feasibility of using liquid gallium as an internal coolant in cutting inserts to effectively remove thermal energy.

## Introduction

The turning of difficult to cut materials is an efficient method for the fabrication of high-quality cylindrical components requiring tight tolerances [1]. In the turning process, the high temperatures created during material removal can result in the thermal ablation of the tool cutting edge, thus increasing the rate of tool wear which invariably impacts the quality of the machined surface and dimensional accuracy. The most common way to address the control of the high temperatures experienced during mechanical cutting has been with the use of external water-based coolants [2–4]. However, there is general recognition that the wide use of these substances has a detrimental effect on the environment once removed as waste disposal [5, 6]. Additionally, the chemical composition itself can have a direct impact on the health of the operator over time [5]. Furthermore, it has been shown that the effectiveness of the coolant in reaching the targeted cutting zone is not optimal [7]. Alternative, more sustainable solutions to the heat problem in subtractive machining, include cryogenic cooling, minimal quantity lubrication (MQL) and internal cooling techniques [5–7]. The last method has shown great promise as a means to transfer thermal energy as a form of sustainable manufacturing.

The potential to employ internal cooling to reduce heat within the cutting tool is drawing the attention of researchers [5, 8–10]. The ability to machine without the use of external liquids, in particular flooding techniques, suggests that dry machining in combination with internal cooling, is an enabling technology which has the potential to realise environmentally friendly machining in industrial applications [5]. To gain an appreciation of how this method can successfully achieve heat transfer, it is useful to conduct a review of existing developments and ascertain the state of the art.

Wu et al. [5], using purified liquid water as the active coolant, constructed an internal channel within an insert and toolholder, which contained an inlet velocity of 10 mm s^−1^ under applied heating conditions of 50 W mm^−2^ over a 1 mm^2^ contact area. The results indicated a temperature reduction at the tool tip of 87.9° C when compared against the same conditions without the coolant applied. Yao et al. [6], designed a closed internal cooling system driven by a mechanical pump in a carbide insert. Using purified water as the active coolant, thermal imaging results indicated a temperature drop from 433.5 to 258.5 °C at the tool tip. Both of these methods employed liquid water as the cooling agent. The limitations are the thermal conductivity and specific heat capacity of liquid water.

Chen et al. [10] used a combinatorial approach in which minimal quantity lubricant was integrated into fabricated internal microchannels of a SiAlON insert and tool holder, fed by a regulated pressurised water–based semi-synthetic fluid. The results showed an 80 °C drop in tool temperature using the combined MQL and internal cooling method when compared with the dry cutting regime. Singh et al. [11] investigated the temperature variance in the tool tip between dry machining and internal cooling with laminar and turbulent flows models. The results revealed the internal cooling design produced a temperature drop of ~ 29% relative to the dry cutting conditions for laminar flow behaviour, and ~ 53% for turbulent flow using the internal cooling method. Shu et al. [12], using a carbide insert, developed a closed internal cooling system for dry machining using liquid water as the coolant. Using an inlet liquid velocity of 0.15 ms^−1^ and an applied heat flux of 20W mm^−2^, the resultant data indicated a maximum temperature reduction of 381.62 to 273.9 °C. Furthermore, it was found that the heat absorption rate (T_eff_-T_max_)/T_ref_ is best at higher coolant circulation velocities. The data suggests that performance of the internal cooling system depends on the geometry of the internal channel along with the velocity and cooling properties of the liquid [12]. Isik et al. [13] fabricated a prototype tool holder with a coated carbide insert consisting of a 2-mm wide internal channel circulating with purified liquid water at 18 °C. Measurements taken over varying cutting speeds indicated a decrease of 9% in tool wear relative to dry machining. Subsequent analysis also revealed a 13% increase in workpiece surface quality (Ra 0.699 µm) of the machine Waspaloy workpiece relative to dry machining. As found in studies by [10], optimum heat transfer and resultant surface quality corresponded to the highest coolant flow rate and cutting speed.

Utilizing a relatively complex design, Öztürk et al. [4] used an in/out liquid water coolant flow that dispersed onto the base of the insert. Then combining with a coolant reservoir, aluminium blocks were used in conjunction with integrated Peltier modules operating as a fan to transfer heat. They compared the data for tool tip temperature between the internal cooling method against dry machining. A 107 °C temperature drop in the tool tip was obtained relative to the dry machining test. Additionally, surface roughness measurements on the 1040 steel workpiece showed values from 0.18 to 2.05 µm.

Neto et al. [8], employed a two-phase pump cooling system in a closed loop design whereby the circulating liquid water vaporises upon contact with a silver interface that acts to cool the cutting tool during machining. Condensing of the vaporised liquid occurred through forced convection at 25 °C, and then was mechanically pumped back at a feed rate of 1.78 min^−1^ into the internal channel system. Experimental data indicated the maximum temperature of the tool with internal cooling was 79 °C lower relative to dry machining conditions.

Uhlmann et al. [9], developed a numerical model of the heat transfer mechanism whereby the heat is conducted through the tool into a copper heat sink. The heat is then transferred through forced convection into a water/water and glycol fluid. The results indicated a temperature reduction of 21% with the water/water glycol agent relative to liquid water. However, this design requires an external pump to circulate the fluid along with a heat exchanger and chiller. As such, it is relatively complex compared to more simple designs.

Shu et al. [14] conducted studies on a closed looped internal cooling design. Using a tungsten carbide insert with embedded thermocouples, experimental results revealed that structural stability of the insert could be maintained with a cutting-edge thickness of 1 mm and a wall thickness of 0.7 mm from the flank face. Numerical modelling revealed that an 82 °C temperature reduction was possible with the design. Overall, the study showed how the effectiveness of the liquid water-cooling system increases as the inlet velocity, heat flux and tool-chip contact area are increased [14].

All of these methods employ liquid water–based coolants which are subsequently mechanically pumped around the internal channel of a cutting tool or/and toolholder. Although this method offers good potential, it is limited as a sustainable cooling process in two ways. Firstly, liquid water has a maximum thermal conductivity of 0.6 W m·K at 20 °C. This poses a question, namely, is there an alternative substance which has a higher rate of heat transfer which can be employed for the same purpose? The second problem with the majority of internal cooling systems, is that it requires some form of mechanical power to generate the energy to provide circulation of the coolant. The second question therefore is whether it is possible to engineer a solution so that the internal liquid within the cutting tool can be pumped around the circuit without the additional power? This study attempts to address these two issues by proposing a novel means to transfer heat in a 3D printed ceramic cutting insert.

Austenitic stainless steel 316L is widely used in the aerospace and biomedical industries due to its high strength, biocompatibility, and corrosion resistance [15, 16]. Despite its widespread use as the material of choice for precision applications, it remains problematic to shape the alloy through traditional subtractive manufacturing processes. This is because of its work hardening and its propensity for built up edge (BUE) formation on the cutting tool, which makes it challenging to selectively remove material into a final form of high quality [17]. Additionally, its low thermal conductivity affects the mechanism of heat transfer within the cutting zone.

Aluminum oxide (Al_2_O_3_) is a class of technical ceramic that exhibits high thermal and wear resistance, high hardness at elevated temperatures, and chemical inertness under most machining conditions [18]. Its unique physiochemical properties (Table 1) allow it to be used under extreme conditions requiring the attributes outlined, it therefore is a suitable choice of material as a cutting tool when machining hard to cut materials such as 316L. Notwithstanding the advantages Al_2_O_3_ has over high-speed steels and carbides, it also suffers from the problem of low thermal conductivity [18]. This means that the heat transfer rate is poor relative to most metal inserts, therefore an alternative method is needed to transport the thermal energy accumulated as a result of the tool-workpiece interaction.

**Table 1:** Properties of sintered Al_2_O_3_ (LithaLox 500) [19]

As sintered	Value
Relative density [%]	98.4
Porosity [%]	1.6
Surface roughness Ra [μm]	0.9
Hardness [HV10]	1450
Thermal conductivity [W/(m.k)]	37
Maximum operating temperature [°C]	1650
Specific electrical resistivity [Ω.cm]	~ 1014
Youngs modulus [GPa]	300
Fracture toughness [MPa.m^1/2^]	4–5
Four-Point bending strength [MPa]	430

This paper develops a numerical model which aims to investigate the potential of an internal magnetohydrodynamic system as an alternative to external coolants when machining 316L. This is developed using an Al_2_O_3_ insert with a geometrically defined internal cooling channel [20]. A detailed simulation model is created which focuses on thermal transfer during machining and uses a two-step approach in developing the heat transfer mechanism through the decomposition of the cutting tool interface problem. This is done by implementing a conjugate heat transfer model and then combining the results with the data obtained from the cutting tool interaction. Subsequently, an experimental model is developed and tested using a custom-made desktop-sized turning machine, in order to validate the numerical model.

Current investigations on internal cooling strategies lead to the following conclusions:

- Internal cooling methods can increase the heat transfer rate in cutting tools, producing a measurable reduction in tool wear and improved workpiece integrity overall.
- Liquid water–based coolants are the primary fluid used, thus limiting thermal conductivity effectiveness.
- To date, researches indicate a mechanical pump or external power source is needed to circulate the liquid around the internal channel of the cutting tool. Cyclic circulation without this external input has not been demonstrated.

Furthermore, the elimination of a need for external power realised via the magnetohydrodynamic drive is novel within this form of application.

### Modelling approach

Numerical modelling provides for a powerful method to design and analyse prototype designs before expensive and timely experimental cutting trials are conducted. Computational models take considerable time to accurately construct and test, so it is important to utilize these tools as effectively as possible to reflect the physical model, whilst removing unnecessary calculations [21, 22]. The degree of complexity required to develop 3D models of the heat transfer process is expansive [23–25]. Therefore, simplifying assumptions are employed to reduce computational time. For that reason, careful consideration is given when constructing accurate models, so that parameters and boundary conditions pertaining to the physical model are reflected in the simulated process.

Finite element method (FEM) is a widely used analytical method to approach the problem from a realistic 3D configuration in terms of how the tool-chip interaction behaves. Typically, cutting processes consider the tool-workpiece couple as a global chip formation analysis involving numerical models [26]. This method analyses tool-chip interaction, cutting forces, and chip morphology. Alternatively, the thermomechanical loading approach is adopted [26, 27]. In this case, the cutting tool itself is considered, and how it behaves under specific machining conditions within appropriate boundary conditions. This includes the tool-workpiece contact area, frictional coefficients and the heat zone, the values of which are taken from actual physical models during experiments [26, 28]. However, this study is primarily concerned with analysis of the heat transfer through the internal coolant during the machining tests, therefore, mechanical machining tests, both simulation and experimental relating to the tool-chip interaction will not be considered.

In modelling the internal channel and the fluidic cooling action of the liquid gallium, it is necessary to integrate the boundary and initial conditions of the system. Process parameters include structural, thermal, and fluid flow analysis. These are conducted in ANSYS Workbench using thermomechanical and FLUENT analysis tools. It is important to note that the purpose of this study is to ascertain the effectiveness of the magnetohydrodynamic cooling system, using the modelling tools as an engineering aid to optimise the geometrical dimensions of the cutting insert. Furthermore, this study is concerned with thermal transfer during the machining process, it is not concerned with entire machining process simulation from a dynamic viewpoint with subsequent experimental validation of the cutting mechanics. Instead, the primary focus is on the heat that is created and transferred through the solid–liquid boundary and dissipated into a heat sink.

### Heat source and the cutting tool

**Fig. 1:**
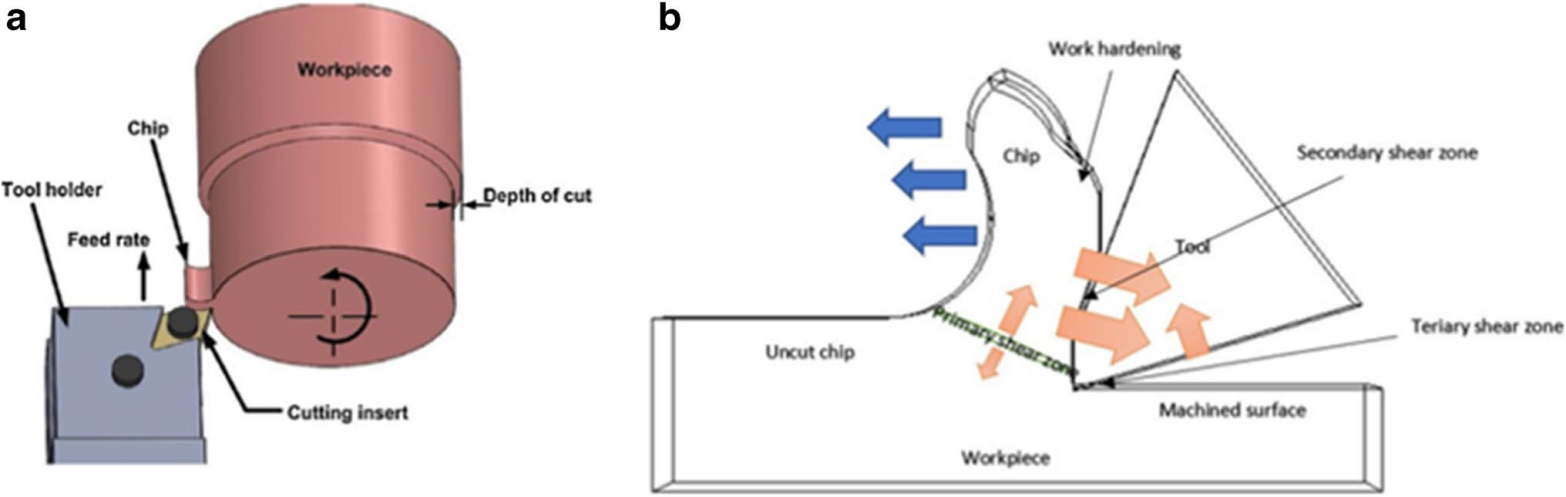
The generation of heat in mechanical cutting. (**a**) CAD Illustration of the orthogonal cutting mechanism in the turning operation [11]. (**b**) Schematic illustrating the mechanism of heat transfer when machining with materials of low thermal conductivity

In mechanical cutting, the three heat source zones are located in the primary shear plane, secondary shear zone and tertiary friction zone as shown in Fig. 1. The cutting zone produces localised high temperatures which are a dynamic transient process. This presents a challenge in terms of measuring the thermodynamic changes between the tool-workpiece interface. There have been two ways to conduct measurement of this mechanism in situ, where the first is the use of integrated thermocouples [26, 29]. The other method is through thermographic imaging of the actual machining conditions in real time [26]. This can give more accurate measurements, but it is also difficult to implement as the dynamic tool-workpiece interaction along with the surrounding physical and thermal process can restrict the use of this method. Nonetheless, this study adopts the latter approach as the magnetohydrodynamic system cannot be fully measured using the integrated thermocouple technique. The numerical model is developed using data obtained from the experimental tests (including heat flux and heat transfer magnitudes), at the tool-workpiece contact zone, combined with material and boundary condition parameters in the construction of the 3D model. Because of the nature of the solid–liquid boundary, where heat is exchanged between the solid Al_2_O_3_ insert walls and the liquid gallium, it is necessary to employ a conjugate heat transfer (CHT) model [30, 31].

## Materials and methods

### Ceramic cutting tools

Ceramic cutting tools are used where high cutting speeds, excellent wear, and heat resistance are required typically on difficult to cut materials [32]. Physical properties and associated physical conditions and requirements relating to ceramic tools are listed in Table 2. Typically, they are used without external coolants. This provides for the cutting tool to maintain the localised thermal energy at the cutting edge, whilst the tool moves across the surface of the workpiece [32, 33]. Ceramic cutting tools require the high thermal energy because the high heat produced in front of the cutting tool creates a plasticisation of the uncut surface, allowing for ease of removal with the heat resistant ceramic tip. Nonetheless, wear occurs, and the excess heat generated can adversely affect tool life and surface finish [34]. Thus, coolants are still required, however, a specific form of heat removal is necessary to prevent thermal shock occurring in the ceramic material [35]. Instead of flooding the cutting zone, a gradual reduction in heat is required to avoid thermal shock. This is why internal cooling is an enabling process, where it can provide a means to remove the excess heat whilst maintaining thermal stability on the ceramic surface.

**Table 2:** Conditions and requirements for ceramic cutting tools [34, 35]

Conditions	Requirements
High thermal load capacity	High hot hardness
High stress/strain rate	Mechanical shock resistance
Dry cutting	Low wear rate
Continuous cutting	Chemical inertness

### Fabrication

**Fig. 2:**
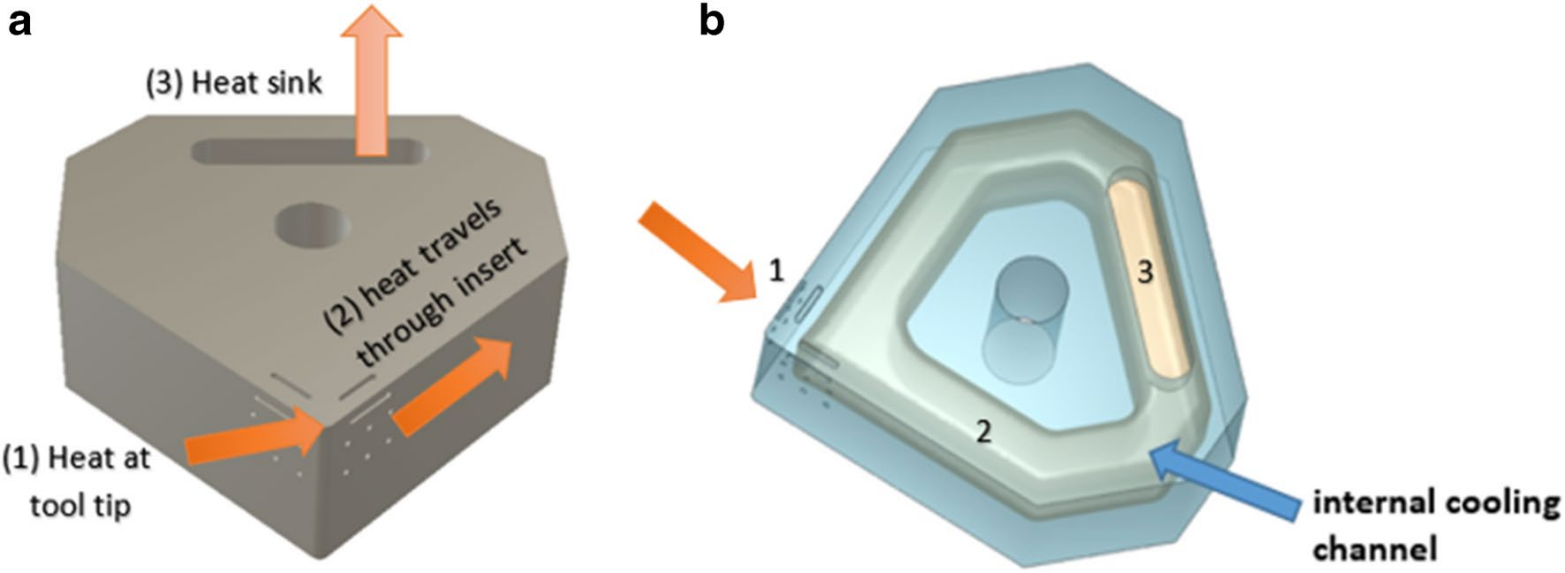
The direction of the heat. (**a**) CAD model of the solid insert showing the primary contact region (1), the flow direction (2) and transfer at the heat sink (3). (**b**) CAD model of the internal channel illustrating the heat transfer process

The ceramic inserts are fabricated using a 3D lithographic ceramic manufacturing (LCM) printer (CeraFab 7500), in which high-purity aluminium oxide (99.98%) photocurable ceramic suspension, LithaLox HP 500, is the forming ceramic slurry. The LCM process provides for manipulation of the CAD model in 3D orientation within a digital platform embedded within the CeraFab 7500 program. Combining this geometric versatility with forming processing parameters including layer thickness, light intensity, and exposure time, provides for tailored modification of the process in situ, and therefore control of the material properties during the forming phase. For this study, a 25-µm layer thickness was employed. Post forming of the green body, the debinding and sintering phase is conducted in an air atmosphere programable electric furnace (Carbolite Gero RHF 1600), using parameters from the manufacturer [36]. A CAD model of the insert with the primary tool-chip contact region and heat flow route are shown in Fig. 2a, with the internal channel visible in Fig. 2b. The geometrical specifications of the cutting insert are given in the Appendix.

### Experimental set-up

**Fig. 3:**
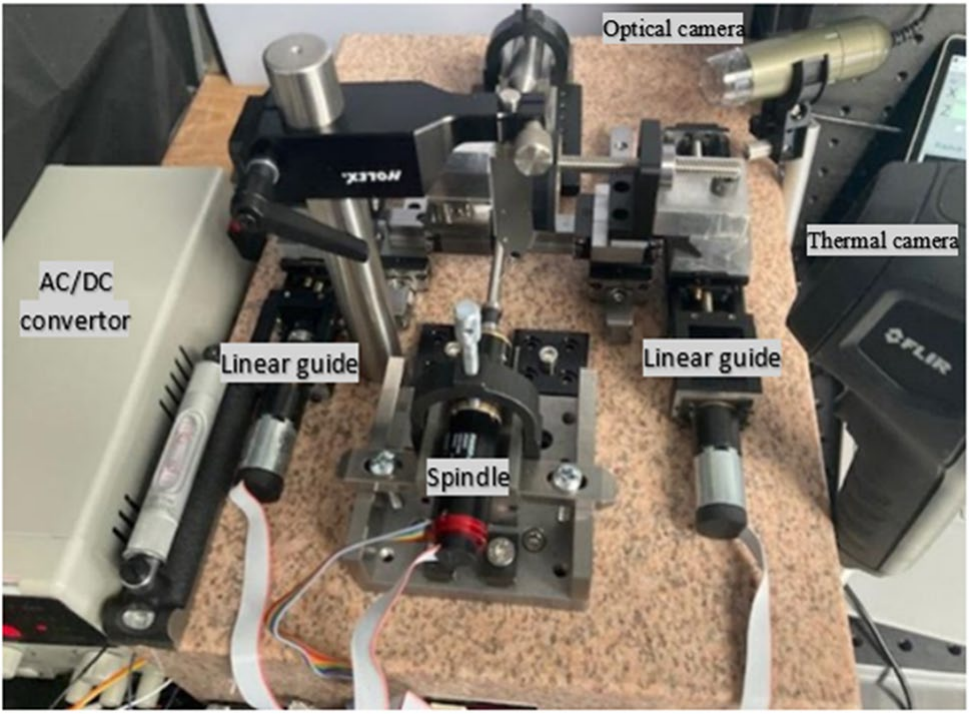
Custom built turning machine

To ascertain the magnitude of the heat flux into the cutting insert an experimental machining test is performed using a custom-built desktop turning machine (Fig. 3). The temperature distribution on the insert is measured using a high temperature thermographic camera (TG297 FLIR^©^) which targets the cutting zone. A series of machining tests are performed on 6 mm 316L cylindrical workpieces under dry conditions. The parameters are listed in Table 3*.* A total of six tests runs are performed with the average value obtained for the temperature distribution series at the cutting edge used in the model. Prior to testing, tool run-out measurement was performed.

**Table 3:** Machining conditions used for the experimental tests

Machining parameters	1	2	3
Cutting speed *V*_*c*_	250 m/min	500 m/min	900 m/min
Feed rate *f*	0.08 mm/rev	0.08 mm/rev	0.08 mm/rev
Depth of cut *a*_*p*_	0.1 mm	0.1 mm	0.1 mm

A pre-measured volume of solid gallium (Table 4) was heated above its glass transition temperature, and then injected into the heat sink chamber of the cutting insert*.* It was then fixed in position atop a tool holder system. The machine tool system was controlled through interfaces linked to a PC using a Mach 3^©^ CNC interface.

**Table 4:** Properties of elemental gallium [37]

Parameter	Magnitude
Density (at 20°)	5900 kg/m^3^
Melting point	29.78 °C
Boiling point	2300 °C
Latent heat of melting	8.03 × 10^4^ J/kg
Linear expansion coefficient (at 20 °C)	1.8 × 10^5^ °C^−1^
Specific heat (at 0–16 °C)	0.373 × 10^3^ J/kg K
Thermal conductivity (at 20 °C)	29.308–37.681 W/m·K
Electrical resistivity	4.5 × 10^–7^ Ohm m
Compressibility coefficient (at 20 °C)	2 × 10^–10^ m^3^/kg
Evaporation pressure	10^–12^ mm/Hg
Surface tension	0.707 N m^−1^

### Heat transfer in the cutting tool

The Al_2_O_3_ cutting tool is a solid material, as such, it obeys Fourier’s law for thermal conduction:

2.1 $$Q= -kA\frac{dT}{dx}$$

where, *Q* is the conductive heat transfer rate, *k* is the thermal conductivity of the cutting insert, *A* is a constant and $\frac{dT}{dx}$ is the thermal gradient. The geometrical specifications of the insert (see the Appendix Fig. 27) provides for a simple geometry, allow for insight to the underlying heat transfer mechanism when developing the numerical model.

The circulation of the fluid (water or gallium) can be considered a form of forced convection, therefore, the heat transfer rate can be solved based on the temperature change with the heat transfer coefficient magnitude. The Newtonian cooling formula for convective heat transfer is given as

2.2 $$q=h\left({T}_{fluid}-{T}_{wall}\right)$$

where, *q* is the convective heat flow rate, *h* is the heat transfer coefficient for convection, *T*_*fluid*_ and *T*_*wall*_ are the fluidic and solid wall temperatures respectively.

It can be seen from Eqs. (2.1) and (2.2) that solid heat transfer depends on the thermal conductivity *k,* whereas the liquid transfer depends on the convective heat transfer coefficient *h.*

### Liquid metal flow within the internal channel

**Fig. 4:**
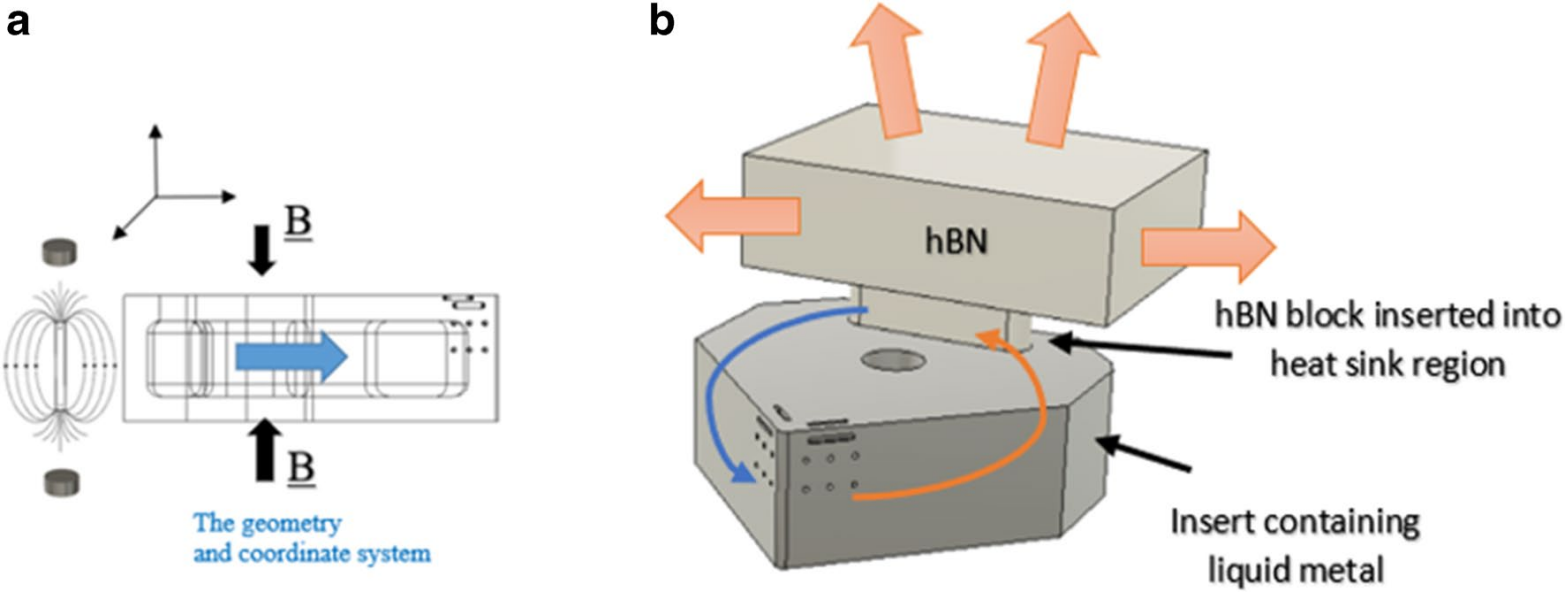
The magnetohydrodynamic process. (**a**) CAD model of the MHD with the neodymium magnets perpendicular to the liquid metal channel. (**b**) Mechanism of thermal transfer through the hBN heat exchanger

A series of neodymium magnets (Eclipse), with a diameter *d* of = 3 mm, and height *h* of = 2 mm, located at fixed distance on opposite faces of the 3D fabricated aluminium oxide insert, generates a homogeneous magnetic field, normal to the liquid metal (elemental gallium 99.8%, Goodfellow) within the channel as shown in Fig. 4a. When an electric current is applied to the liquid metal it modifies its dynamic motive fluidic behaviour, creating an electromotive force known as the Lorentz force within the circulating liquid. For the model, it is assumed the liquid metal is electrically conducting, incompressible and in a steady state within the dimensions of the channel. The magnetic field on the surface of the insert is ± *B*_*O*_*e*_*Z*_ with a magnitude of ~ 1.2 T (the magnetic field strength of the neodymium magnets)*.* Using the value *B*_*o*_ to represent the applied magnetic field, the magnitude is given by:

2.3 $${H}_{a}= {B}_{o}H\sqrt{\left(\frac{\sigma }{\rho v}\right)}$$

where *H*_a_ is the dimensionless Hartmann number describing the ratio of electromagnetic forces to viscous forces, $\sigma$ is the electric conductivity, *ρ* the density and *v* the velocity. It is assumed that the liquid gallium behaves as an incompressible fluid with velocity *U*_*O*_, then applying the ratio of inertial and viscous flow in a liquid gives,

2.4 $${R}_{e}=\frac{{U}_{o}H}{v}$$

where *Re is* the dimensionless Reynolds number with *v* the kinematic viscosity. The Reynolds number (in this case) is primarily of laminar flow behaviour within the MHD system, resulting in stable flow currents along the wall boundaries of the internal channel. As the Al_2_O_3_ insert is electrically insulating, the solid domain in the problem is zero. Finally, applying the geometric parameters defined, along with the values for *R*_*e*_,* H*_*a*_, and the physical properties of elemental gallium (Table 4) provides the boundary conditions used in the model. From a thermal perspective, liquid metals differ from other liquid media due to their higher thermal conductivity λ, and lower specific heat capacity c_p_ [37, 38]. These two parameters can be combined into a dimensionless number used in fluid dynamic heat transfer problems, called the Prandtl number (Pr). This number represents the ratio of momentum diffusion to thermal transport in a fluid, given by:

2.5 $${\text{Pr}}= \frac{pv{c}_{p}}{\lambda }= \frac{v}{k} , k= \frac{\lambda }{p{c}_{p}}$$

where *C*_*p*_ is the specific heat capacity, ρ is the density, λ the thermal conductivity and *k* the thermal diffusivity. Liquid gallium has a Prandtl number of ≈ 0.028.

### Heat sink

**Fig. 5:**
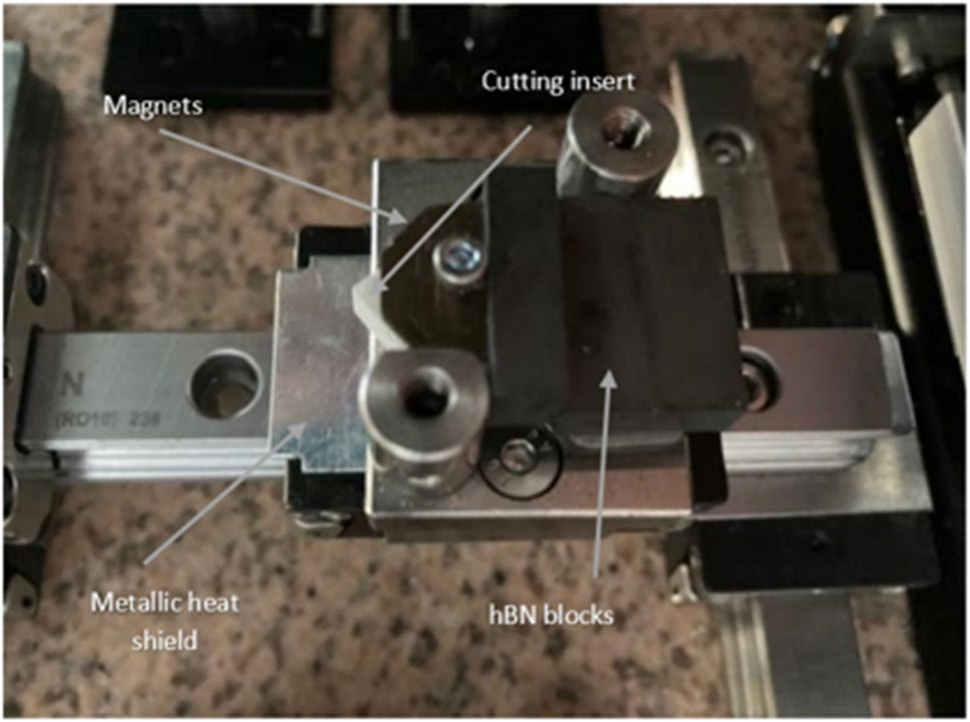
Image of the MHD coolant system [20]

A heat sink is required for the MHD system (Fig. 4b). This is done to address the limitations of the finite heat capacity of the Al_2_O_3_ insert and the liquid metal within the dimensions of the internal channel. This introduces a limiting factor in the form of transient thermal equilibrium, at which point the temperature differential has effectively stopped, this means heat can no longer be transferred. Therefore, the introduction of the heat sink provides for transfer of the accumulated thermal energy which is then exchanged with the ambient air. Hot pressed hexagonal boron nitride (hBN) was selected due to its compatibility with liquid gallium in terms of wettability and corrosion resistance and its superior thermal conductivity performance. A custom made hot pressed hBN component, which matches the local geometry of the insert heat sink coupling, is fixed in position as a thermal exchanger. The physical prototype is shown in Fig. 5*.* The dimensions of the hBN heat sink (L × W × H) are 15 mm × 10 mm × 4.5 mm.

## Numerical modelling

### Conjugate heat transfer model

#### Governing equations for conjugate heat transfer

The conventional approach of heat transfer analysis through isothermal or constant heat flux boundary conditions do not give accurate predictions in real life coupled heat transfer problems [30]. Accurate models require the coupled solution of governing equations in the fluid and solid interfaces in terms of temperature distribution and heat load on the solid surface. This method is known as conjugate heat transfer (CHT) analysis and will be the approach adopted in modelling the thermal energy through the solid–liquid boundary in the ceramic cutting tool. The most crucial parameters required for the analysis of heat transfer problems are the local temperature, heat transfer coefficient of the surface and the heat flux [30, 39]. These form part of the boundary conditions, in which the heat is absorbed or transferred from the solid body across to the fluid or vice versa. The challenge in solving this problem through analytical methods is partly due to the transient nature of the process which is highly complex [30, 40]. To circumvent this, a conjugation of the boundary condition at the solid–liquid phase through coupled heat transfer analysis is employed via a numerical method. This forms the basis of the thermal energy transfer analysis during the motion of the liquid gallium and liquid water and is therefore representative of the cooling mechanism used in this study. The development of the governing equations are approached in a two-phase method.

The process applies the following assumptions:

- Thermomechanical analysis is performed using FEA on the cutting tool and workpiece interaction with subsequent measurement of the temperature distribution on the cutting tool.
- Heat sources are a reflection of the physical model, namely the primary deformation, secondary deformation and frictional contact area.
- Material properties, boundary and initial conditions are taken from the physical model. This is simplified as deemed necessary by computational time restrictions but retains realistic values to be an accurate reflection of the system.
- The CHT model is employed for the solid–liquid boundary interfaces.
- Comparison of the CHT model is with liquid water against liquid gallium only.
- The same conditions and parameters in respect to the physical inputs are applied to both fluids.
- The cutting speed is the only variable that changes in the experimental model. The feed rate and DOC are fixed (Table 3). This is to limit the range of variables that can affect the process and allow for simplified comparison.

The internal liquid water model is restricted to the simulation test only: The experimental model is designed for the magnetohydrodynamic system and therefore cannot be used to compare in this case. However, the simulation model remains a valid comparison as the liquid gallium simulation is performed using identical boundary conditions.

The system uses a magnetohydrodynamic drive and does not require mechanical power to circulate the fluid around the inner channel. However, for the model, it is not feasible to construct a magnetohydrodynamic drive into FEA analysis as this would be prohibitively long and extremely complex to solve numerically. Therefore, a substitute was used, whereby the circulation velocity of the liquid metal is based on the actual experimental tests on the applied magnetic field when active. This means that the model is a good reflection of the actual system and gives confidence in its accuracy. This is an acceptable compromise as the fluidic motion considers the material thermal properties once dynamic motion is initiated, and this is included in the inputted parameters.

#### Phase 1: Heat transfer within the cooling channel

To develop the formula required to analyse the internal cooling mechanism, it is instructive to first define the case where there is no internal cooling, in this case, heat conduction through the Al_2_O_3_ body is only considered. This can be expressed in Cartesian coordinates as

3.1 $$\frac{{\partial }^{2}T}{\partial {x}^{2}}+\frac{{\partial }^{2}T}{\partial {y}^{2}}+\frac{{\partial }^{2}T}{\partial {z}^{2}}= \frac{\rho c}{k}\frac{\partial T}{\partial t}$$

where *ρ* is the mass density, *c* the heat capacity, *k* the heat conductivity coefficient and *t,* time. This is not adequate however for when internal cooling is present. As the fluid is within the internal channel, it therefore necessitates a convective heat transfer model. The heat absorbed by the fluid can be expressed as

3.2 $$Q= {A}_{i}{\rho }_{p}{V}_{i}{C}_{p}\left({T}_{1}-{T}_{2}\right)$$

where *A*_*i*_ is the area inlet for the fluid, *ρ*_*p*_ is the fluid mass density, *V*_*i*_ is the fluid velocity and *C*_*p*_ the heat capacity of the fluid. *T*_*1*_ is the temperature of the fluid going into the channel and *T*_*2*_ the temperature of the fluid going out.

Using Newton’s Law of cooling, the foundations for the equations are encapsulated within the conservation of mass, momentum, and energy laws. Treating each law separately, then for mass conservation, fluidic mass is also conserved which can be described by the following equation:

3.3 $$\frac{\partial \rho }{\partial t}+\nabla \bullet \left(\rho \mu \right)=0$$

where, *ρ* is the fluid density, *µ* is the flow velocity vector field, and *t,* the time which is a local relationship known as the differential form of the continuity equation. This states that rate of mass into a system is equal to the rate of mass out of a system (including additional mass within the system). However, as the fluid in this case (water, gallium) is considered incompressible, then this can be simplified to

3.4 $$\nabla \bullet \mu =0$$

which is a volume continuity equation. Physically, this means that as the fluid transverses through a channel, any variation in diameter will produce an increase or decrease in local fluid velocity.

Momentum conversation is based upon Newton’s second Law of motion which can also be stated as the Navier–Stokes equation. This equates the rate of change of momentum to applied forces. In cartesian coordinates this is expressed as:

3.5 $$\rho \left(\frac{\partial u}{\partial t}+u\frac{\partial u}{\partial t}+v\frac{\partial u}{\partial t}+w\frac{\partial u}{\partial t}\right)=-\frac{\partial \rho }{\partial x}+u\left(\frac{{\partial }^{2}u}{\partial {x}^{2}}+\frac{{\partial }^{2}u}{\partial {y}^{2}}+\frac{{\partial }^{2}u}{\partial {z}^{2}}\right)+{F}_{x}$$

3.6 $$\rho \left(\frac{\partial v}{\partial t}+u\frac{\partial v}{\partial t}+v\frac{\partial v}{\partial t}+w\frac{\partial v}{\partial t}\right)=-\frac{\partial \rho }{\partial y}+u\left(\frac{{\partial }^{2}v}{\partial {x}^{2}}+\frac{{\partial }^{2}v}{\partial {y}^{2}}+\frac{{\partial }^{2}v}{\partial {z}^{2}}\right)+{F}_{y}$$

3.7 $$\rho \left(\frac{\partial w}{\partial t}+u\frac{\partial w}{\partial t}+v\frac{\partial w}{\partial t}+w\frac{\partial w}{\partial t}\right)=-\frac{\partial \rho }{\partial z}+u\left(\frac{{\partial }^{2}w}{\partial {x}^{2}}+\frac{{\partial }^{2}w}{\partial {y}^{2}}+\frac{{\partial }^{2}w}{\partial {z}^{2}}\right)+{F}_{z}$$

Conservation of energy applied to a fluid in motion equates the rate of change of energy in the fluid to the sum of the net heat flux into the fluid, and the rate of work done on the fluid by the applied forces. This can be expressed as:

3.8 $$\rho {C}_{p}\left(\frac{\partial T}{\partial t}+u\frac{\partial T}{\partial x}+v\frac{\partial T}{\partial y}+w\frac{\partial T}{\partial z}\right)=\varnothing +\frac{\partial }{\partial x}\left[k\frac{\partial T}{\partial x}\right]+\frac{\partial }{\partial y}\left[k\frac{\partial T}{\partial y}\right]+\frac{\partial }{\partial z}\left[k\frac{\partial T}{\partial z}\right]+\left(u\frac{{\partial }^{2}\rho }{\partial {x}^{2}}+v\frac{{\partial }^{2}\rho }{\partial {y}^{2}}+w\frac{{\partial }^{2}\rho }{\partial {z}^{2}}\right)$$

#### Phase 2: The heat flux generation and thermal transfer using the internal cooling system

Numerical modelling of the heat flux into the cutting tool is performed using a CHT analysis. This is because of the solid–liquid boundary which exists to transfer the thermal energy during machining, and thus requires consideration of a conductive and convective heat approach. Because the internal channel spans the entirety of the insert, it is necessary in this case to consider the entire Al_2_O_3_ insert for analysis. Experimental results obtained from the machining tests are used for the heat transfer problem and therefore provides the required data in the model.

There are three boundary conditions applied:

(1) Applied heat flux at the tool-chip contact area represented through frictional forces only.

3.9 $$Q\rightarrow tool=H_{chip}-H_{tool}\;(T_1-T_2)$$

(2) Heat through the tool: solid–liquid boundary problem solved through a CHT model

3.10 $$Q\rightarrow\frac{solid}{liquid}=H_{tool}-H_{liquid}\;(T_3-T_4)$$

(3) Heat exchange between the liquid–solid boundary through the hBN heat sink

3.11 $$Q\rightarrow\frac{liquid}{sink}=H_{liquid}-H_{heatsink}\;(T_5-T_6)$$

### Conjugate heat transfer procedure

**Fig. 6:**
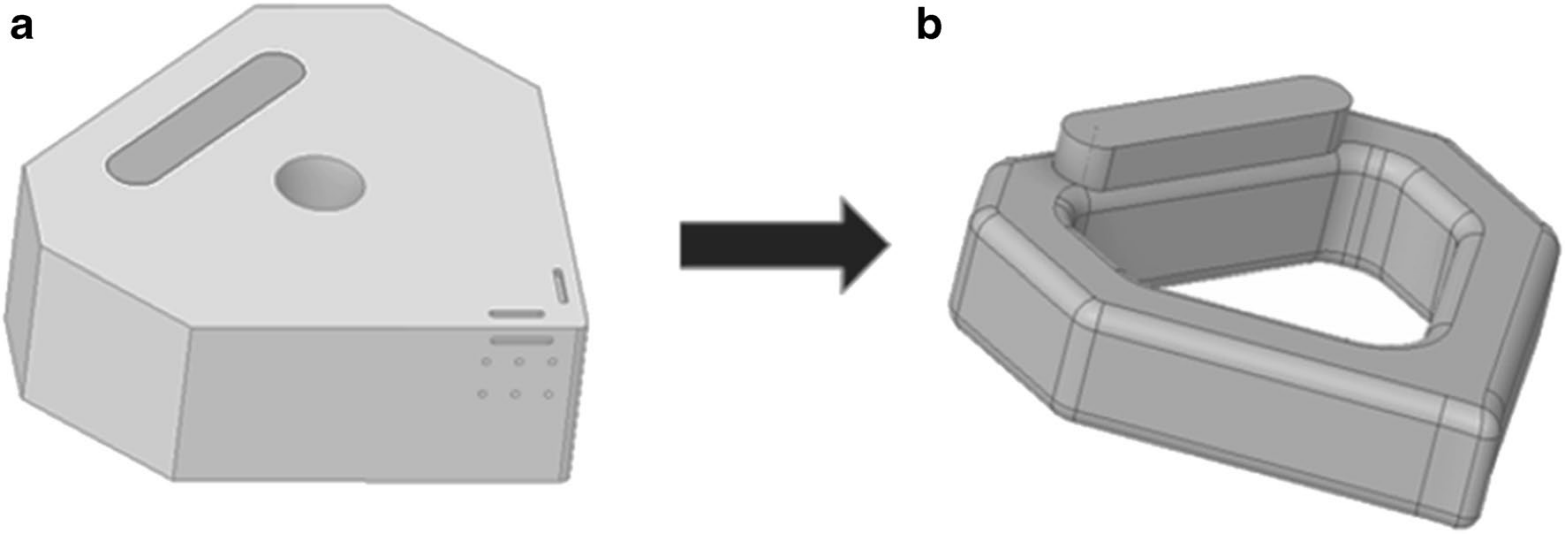
CHT procedure (**a**) Al_2_O_3_ insert with internal channel. (**b**) Extraction of the fluidic region

CHT analysis is performed by extracting the predefined inner channel (Fig. 6). The machining conditions (temperature, heat flow, etc.) and material properties used in the thermomechanical model were applied to the insert, with the boundary conditions and solid-fluidic parameters created within the FLUENT tool (Tables 5, 6 and 7).

**Table 5:** Al_2_O_3_ properties used in the CHT model [20]

Property	Value
Relative density [%]	98.4
Hardness [HV10]	1450
Thermal conductivity [W/(m·k)]	37
Maximum operating temperature [°C]	1650
Specific electrical resistivity [Ω.cm]	~ 1014
Relative permittivity	9.8–10
Youngs modulus [GPa]	300
Fracture toughness [MPa.m^1/2^]	4–5

**Table 6:** Simulation parameters for the Al_2_O_3_ insert

Parameter	Magnitude
Heat flux	15 W/mm^2^
Heat source location	Cutting edge of insert
Heat transfer coefficient of outside surface	8 W/(m^2^ K)
Heat transfer coefficient between tool and liquid	25 W/m K
Environment temperature	20 °C
Inlet/Outlet diameter	1.5 mm

**Table 7:** Simulation parameters for the fluids

Parameter	Water	Gallium
Density (20 °C) [kg/m^3^]	998.2	5900
Thermal conductivity [W/m·k] (20 °C)	0.60	37.68
Specific heat capacity [J/(kg·K]	4182	373
Viscosity [Pa·s]	0.0010	0.010
Reference temperature [°C]	20	30
Boiling point [°C]	100	2300

The standard shear-stress-transport (SST) k-ω model is used. For the tool tip temperature, the value is taken from the three ranges obtained experimentally over the three cutting speeds *V*_*1*_, *V*_*2*_, *V*_*3*_ as shown in Table 3, with the resultant temperatures being 300 °C, 500 °C and 600 °C respectively. The applied temperature distribution is radially directed away from the tool cutting edge reducing in temperature according to increasing distance. The ambient air at 20 °C acts as the second fluid boundary with the physical properties incorporated into the model. Two sets of simulation studies are then generated. The first model applies liquid water as the internal coolant over a period of 20 s with the corresponding data extracted from the resultant CHT output results. The second simulation applies the data of the MHD model and integrates these into the liquid gallium conditions to obtain the results for the second CHT analysis for liquid gallium over 20 s. Both sets of data are then formatted accordingly and then compared.

## Simulation results

### Heat transfer simulation

**Fig. 7:**
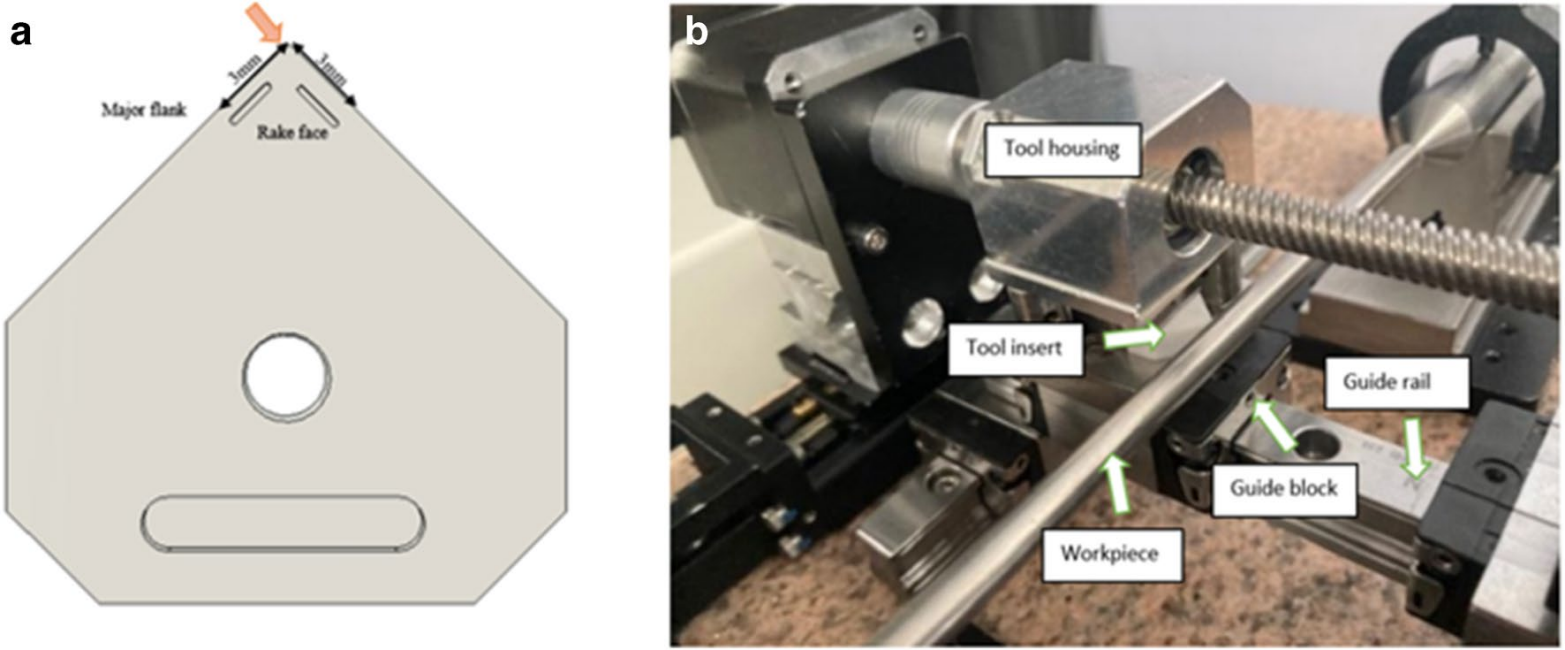
Heat in the cutting tool. (**a**) CAD model illustrating the region of the applied heat used in the simulation. (**b**) Corresponding image of the cutting insert used in the experiment

In the simulation, the local region of tool-workpiece contact is used as the area for heat distribution analysis across the insert (Fig. 7a). This corresponds to the rake and major flank which is the primary region for high temperature exposure during the cutting process in the turning operation [11]. Figure 7b shows the corresponding physical prototype used in the experiment.

**Fig. 8:**
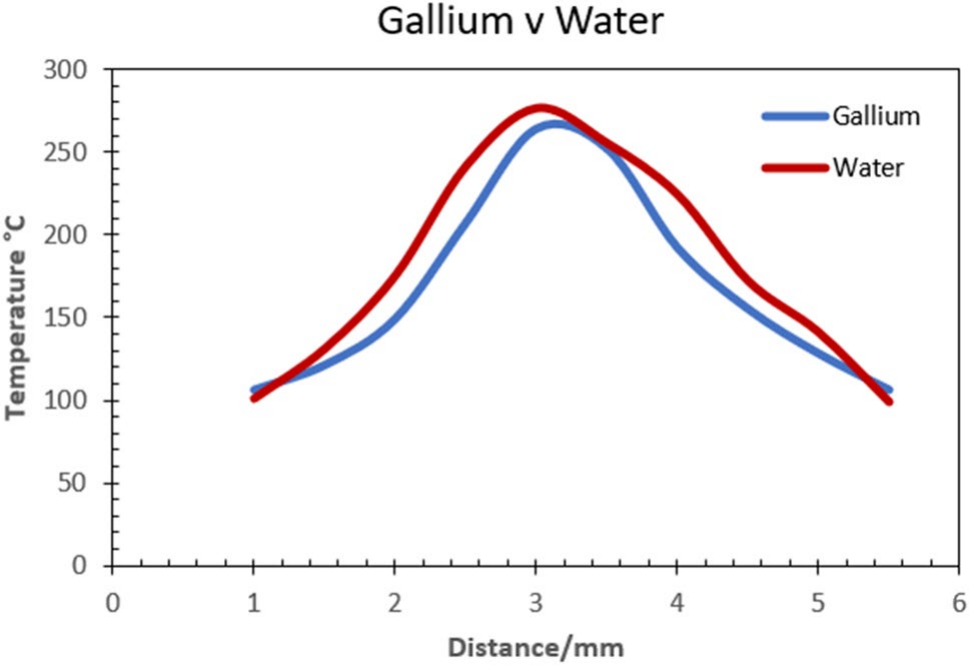
Plot of temperature distribution at the tool edge region with an applied temperature of 300 °C

In Fig. 8, a plot of the local temperature distribution within the internal channel for liquid gallium and liquid water is represented. The data corresponds to a thermal input of 300 °C on the cutting edge region on the insert geometry as shown in Fig. 7a.The results show a temperature difference of 12 °C between liquid gallium and liquid water at the peak values of 276 °C and 264 °C. However, the largest difference is observed along the flank edge at 241 °C and 207 °C which indicates a drop of 34 °C. It is worth noting from Fig. 8 that at the distance of approximately 1 mm, 3.4 mm and 5 mm, the local temperature plots for the two liquid have similar magnitudes. This is likely due to the lower cutting speed of *V*_*1*_ = 250 m m^−1^, which influenced the heat transfer rate of both fluids. At the lower cutting speed, liquid gallium appears to have similar heat transfer rates at the lower temperature regions of the insert relative to the distance measured in the simulation.

The temperature drop from the inputted heat flux and the liquid in the internal channel is accounted for by the dissipation of thermal energy through the Al_2_O_3_ solid and across the boundary into the liquid phase. At this point, the CHT analyses both the solid and fluid using the properties, parameters, boundaries, and initial conditions applied during the development of the model. Therefore, the results are a reflection of those machining conditions which have been modelling on actual data obtained from preliminary experimental machining tests.

The shape of the plot shows that the largest difference in heat transfer occurs along the major flank up to the cutting edge of the insert. This is the region which experiences most temperature exposure during the turning process, so the result it is indicative of what should be expected at this area.

**Fig. 9:**
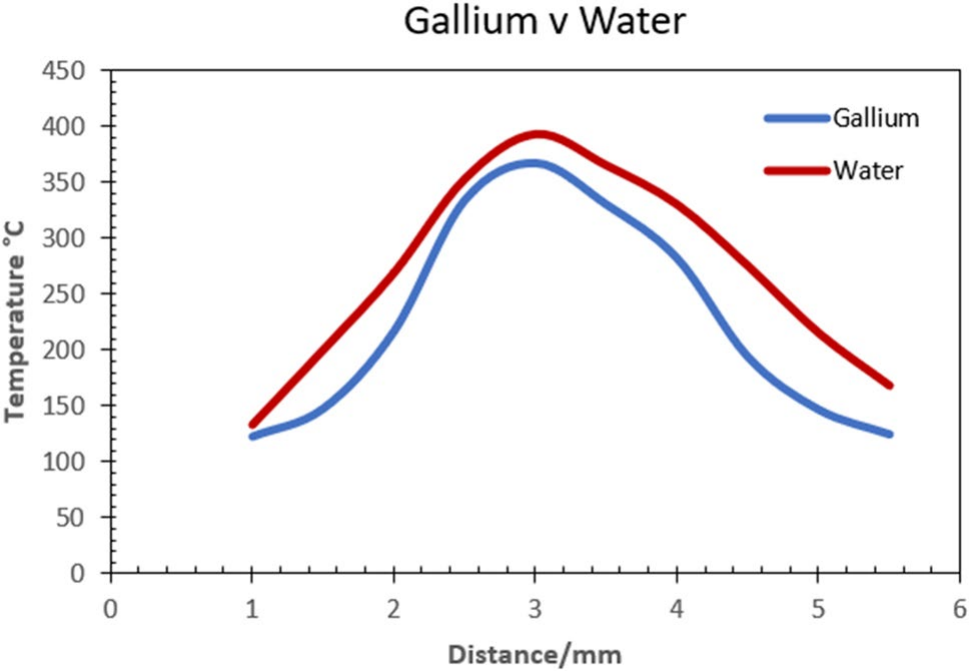
Plot of temperature distribution at the tool edge region with an applied temperature of 500 °C

**Fig. 10:**
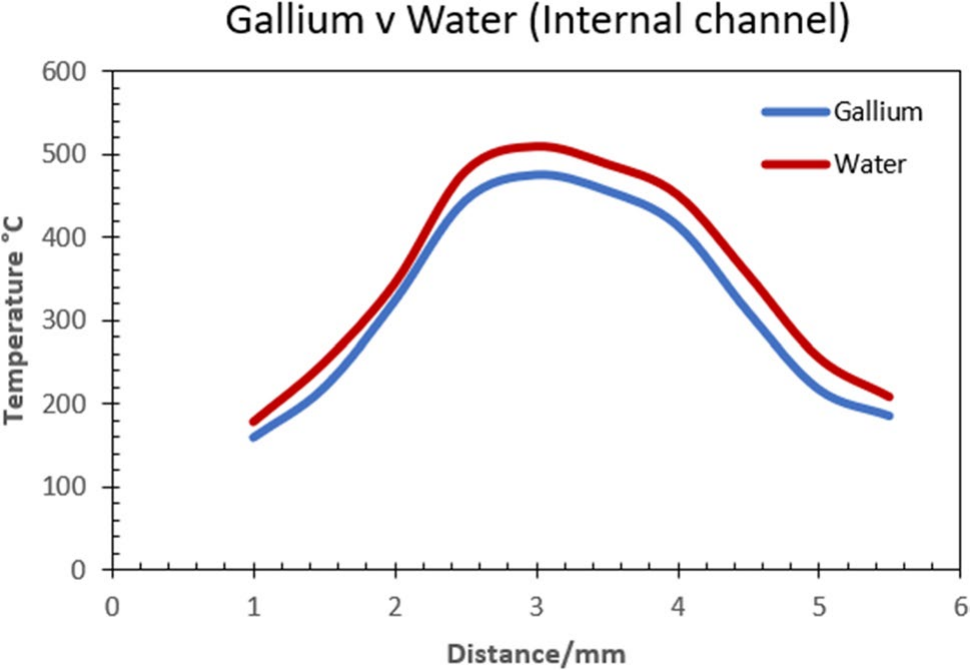
Plot of temperature distribution at the tool region with an applied temperature of 600 °C

The second CHT model used an applied cutting temperature of 500 °C. This magnitude was obtained from experimental data during preliminary testing as before. The resultant plot (Fig. 9) shows the peak values with 367 °C for liquid gallium and 393 °C obtained for liquid water. The temperature difference at the peak values is 26 °C. In this case, the heat transfer is more pronounced at the upper flank away from the cutting edge and surprisingly, larger again at the minor flank outer region within the 3-mm boundary. The final CHT model used an applied cutting temperature of 600 °C. The magnitude was obtained from experimental data during preliminary testing as before. The resultant plot shows a temperature difference of 34 °C, with the peak values of 476 °C obtained for liquid gallium and 510 °C for liquid water (Fig. 10). Examination of the plot shows heat transfer is more pronounced at the cutting edge, with lower heat transfer at the major flank. However, there appears to be consistency in the temperature distribution across the region of the cutting edge and onto the minor flank, which exhibits a similar plot profile for both fluids at different temperatures reflecting the magnitude of heat transfer.

### Comparison of surface cooling between fluids

**Fig. 11:**
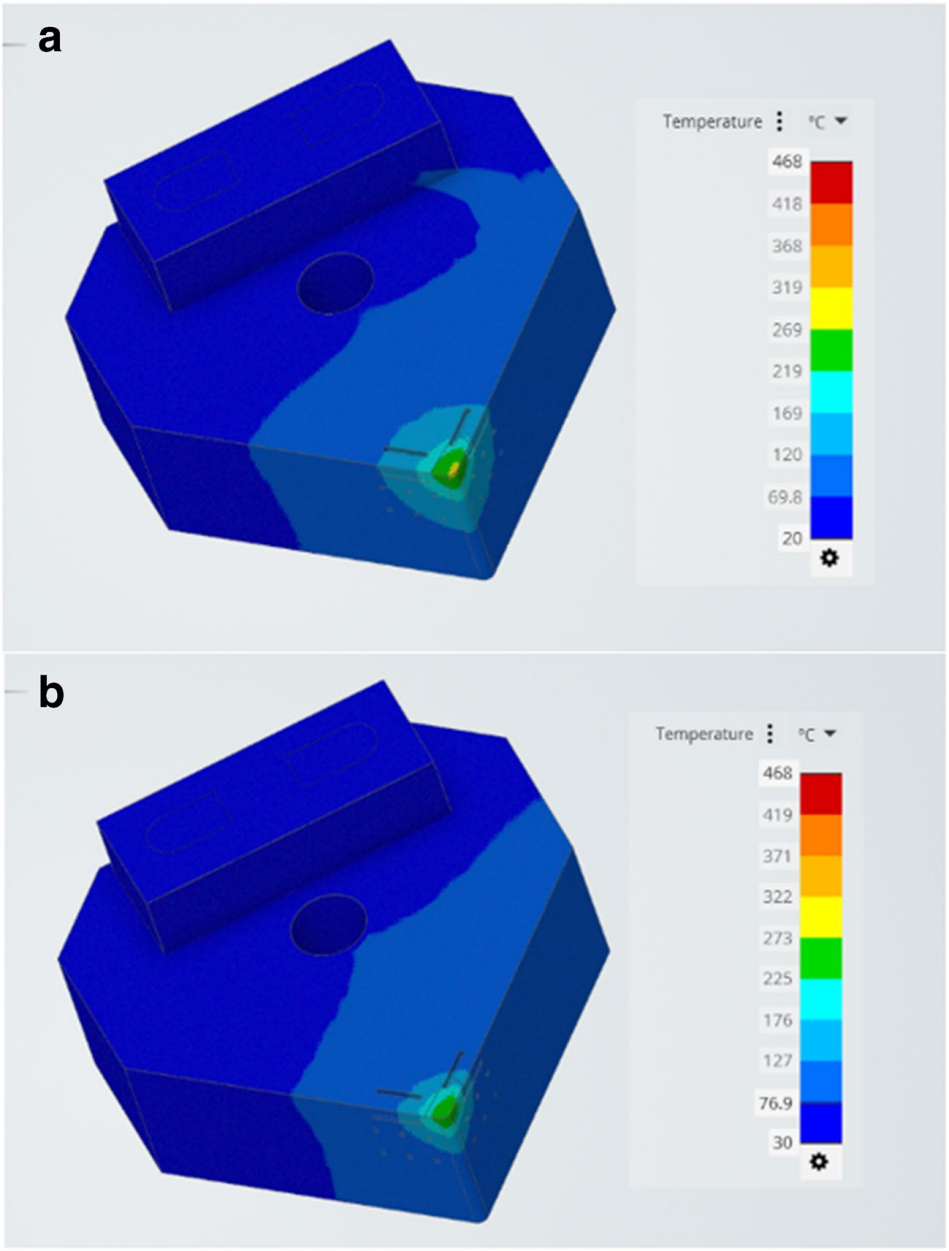
Surface cooling in the model. CHT simulations of the heat transfer for (**a**) liquid water and (**b**) liquid gallium on the exterior surface of the Al_2_O_3_ insert at an applied temperature of 300 °C

The simulation results relate to the fluidic dynamics of the liquid water and liquid gallium when a range of three heat flux regimes (the cutting speeds) are applied under the given parameters in the model. It is useful to visualise the difference on the solid surface of the insert under a single heating regime and compare the temperature difference. Figure 11 is essentially an image of the entire geometry of the cutting insert displaying the heat transfer results from the CHT simulations.

The resultant images show that the liquid gallium outperforms liquid water in terms of heat transfer efficiency relative to surface temperature measurements in the simulation model. The image shows that although the liquid water result (Fig. 11a), shows a further reduction in the surface temperature globally spreading out (represented by the lighter blue colour). Its cutting edge temperature is higher than that of the liquid gallium (Fig. 11b). The liquid gallium is superior at transferring the heat when higher temperatures are present.

**Fig. 12:**
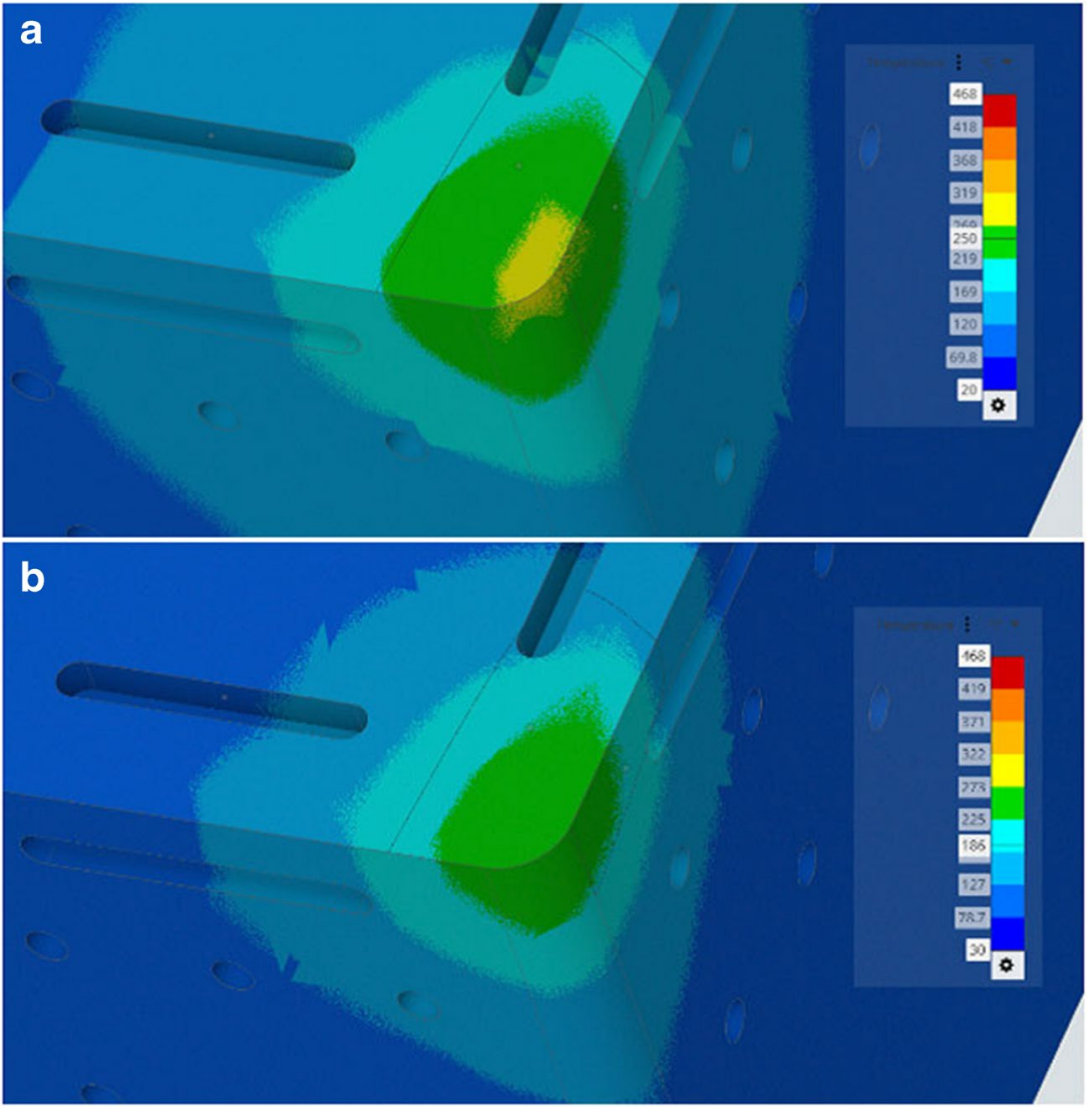
CHT surface temperatures for (**a**) liquid water and (**b**) liquid gallium at 300 °C

Figure 12a shows the corresponding temperature contour given across the cutting tool surface for liquid water. The yellow region is the area of localised highest temperature with a peak value of 311 °C. In Fig. 12b, the temperature range for liquid gallium is again lower as indicated by the reduced temperature under the same conditions at 271 °C. This indicates a surface temperature difference at applied temperature of 300 °C (edge) of 40 °C.

### CFD post processing of the simulation results

To gain better understanding into the heat transfer mechanism, the velocity vectors within the internal channel for liquid water and liquid gallium are measured at a region between the cutting edge and major flank. This region is then extracted for analysis using the FLUENT CFD post processing tool. This function is embedded within the FLUENT program and provides for detailed examination of the simulation results.

**Fig. 13:**
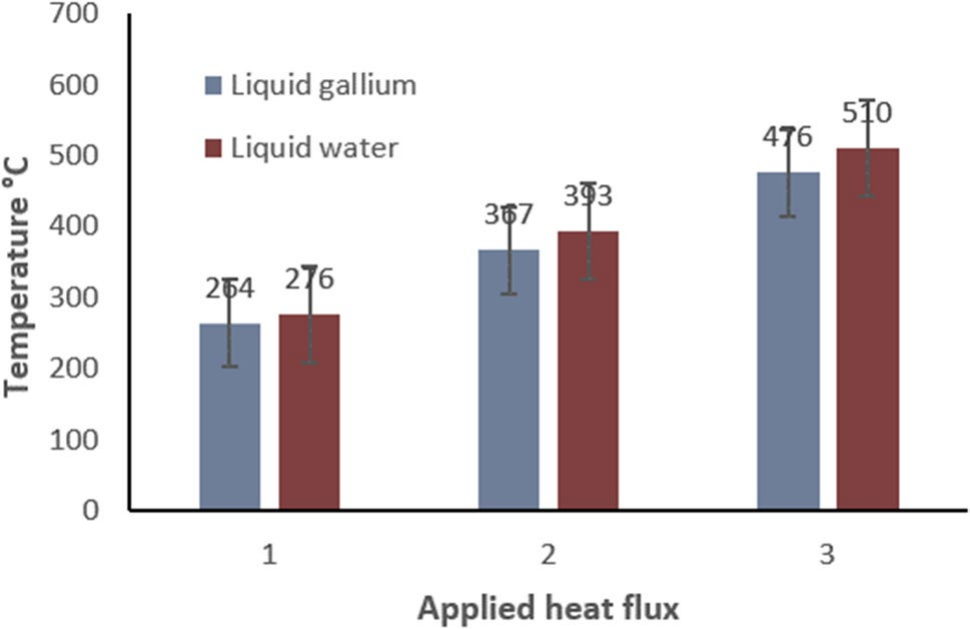
Temperature at the cutting edge for liquid water and liquid gallium. Key: 1 = 250 m min^−1^, 2 = 500 m min^−1^, 3 = 900 m min^−^.^1^ [20]

In Fig. 13, the graph reveals a marked difference between the two fluid performances under the exact same conditions. A nearly linear distribution exists between the two sets of data under the same conditions within the internal channel. This can be attributed to the better heat transfer properties of liquid gallium which results in a reduced surface heat contour effect.

### Velocity flow rates

**Fig. 14:**
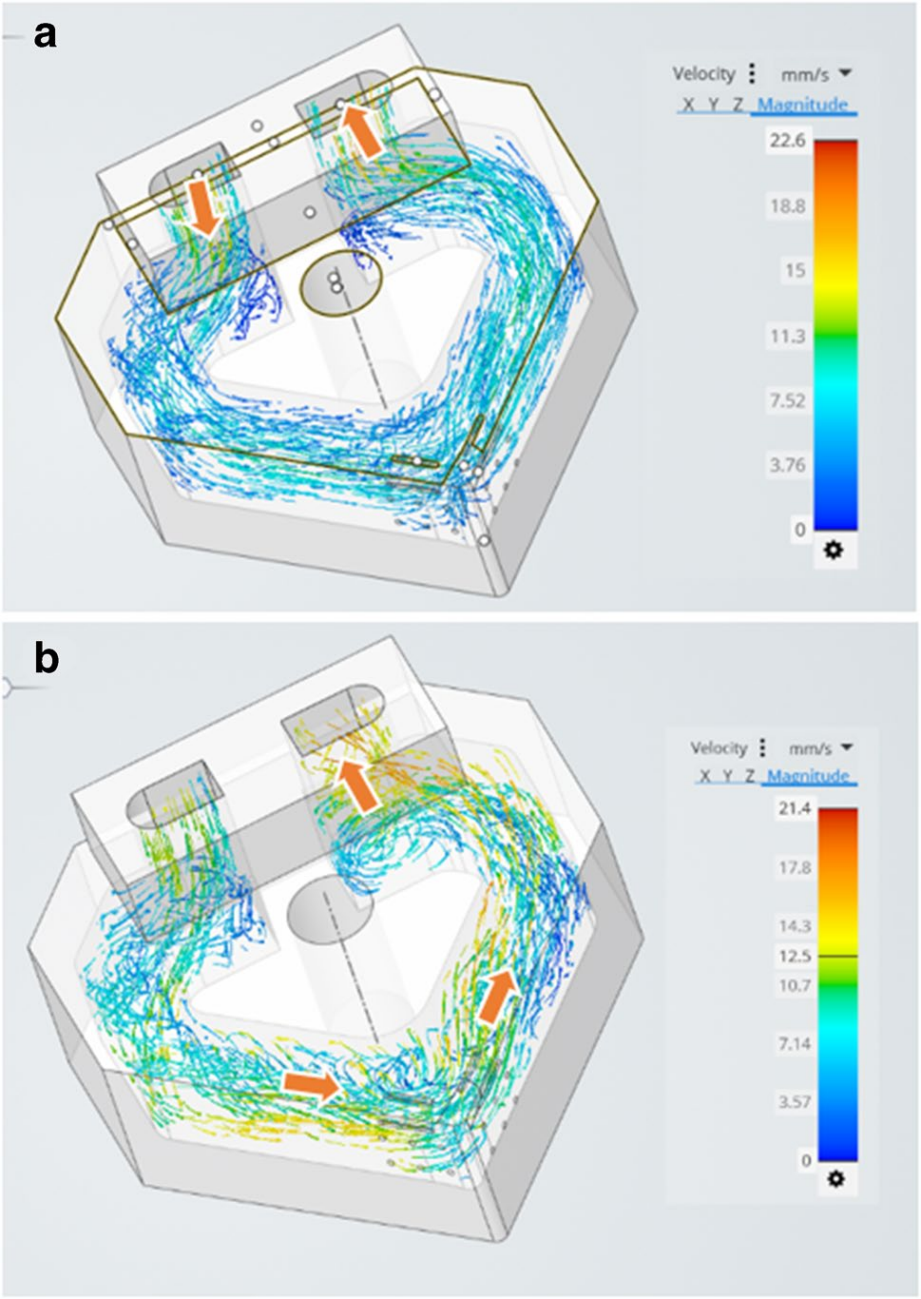
Velocity vectors for (**a**) liquid water and (**b**) liquid gallium under the applied heat fluxes. The arrows represent the areas of highest velocity vector streamlines

Figure 14a shows the velocity profile for liquid water under the application of an applied heat of 600 °C at the cutting edge. The velocity streamlines show that the largest magnitude occurs at the inlet and outlet regions of the internal channel. Contrast this with the velocity streamline obtained for liquid gallium under the same conditions (Fig. 14b). Here, the largest magnitude is at the outlet region followed by the flank areas. This indicates that the fluidic behaviour of the liquid gallium is different from that of water, insofar as the gallium appears to increase its velocity profile where there is a larger temperature difference between the solid interface. This is related to its superior thermal conductivity which affects its streamline velocity within the internal channel when a heat flux is applied.

## Experimental results

Unlike the simulation model, the prototype tool is designed only to measure the effectiveness of the liquid gallium MHD coolant system under controlled conditions using the fabricated cutting insert. Firstly, the Al_2_O_3_ cutting tool is tested under dry machining conditions, followed with the MHD coolant activated. Secondly, the MHD cooling system is then compared against external cooling using liquid water. A thermal imaging camera recorded the cutting process allowing for in situ measurement of the surface temperature variation.

Prior to thermographic measurements, the thermal camera was calibrated to the suitable emissivity levels for ceramic materials. As per initial tests, the workpiece was checked for tool run-out to reduce systematic errors. Three different cutting speeds are used in the trails with the feed rate and DOC kept constant (Table 3). The exact same parameters are used for the insert with the MHD cooling on and off. This was to ensure a fair measurement is obtained and limited to the cutting speed variation only. This restriction allows for analysis of the heat transfer, which is dependent on the Al_2_O_3_ material thermal properties [17, 30], the circulating liquid gallium, and the distance between the outer and inner regions of the internal channel. For each machining test, the tool engaged the workpiece at the predetermined distance (DOC) with the cutting speed controlled though the PC interface (Mach 3®). The length of the tool-workpiece engagement is 100 mm.

### Heat transfer

It was necessary to remove the aluminium housing unit to enable the target area to be measured with the laser imager in the camera. This meant that the *y*-direction was inoperable and by extension fixed the DOC. However, as the DOC was intended to be fixed for the test along with the feed rate, this was acceptable. This was the only way in which the target area (the cutting zone) could be imaged without visual obstruction.

**Fig. 15:**
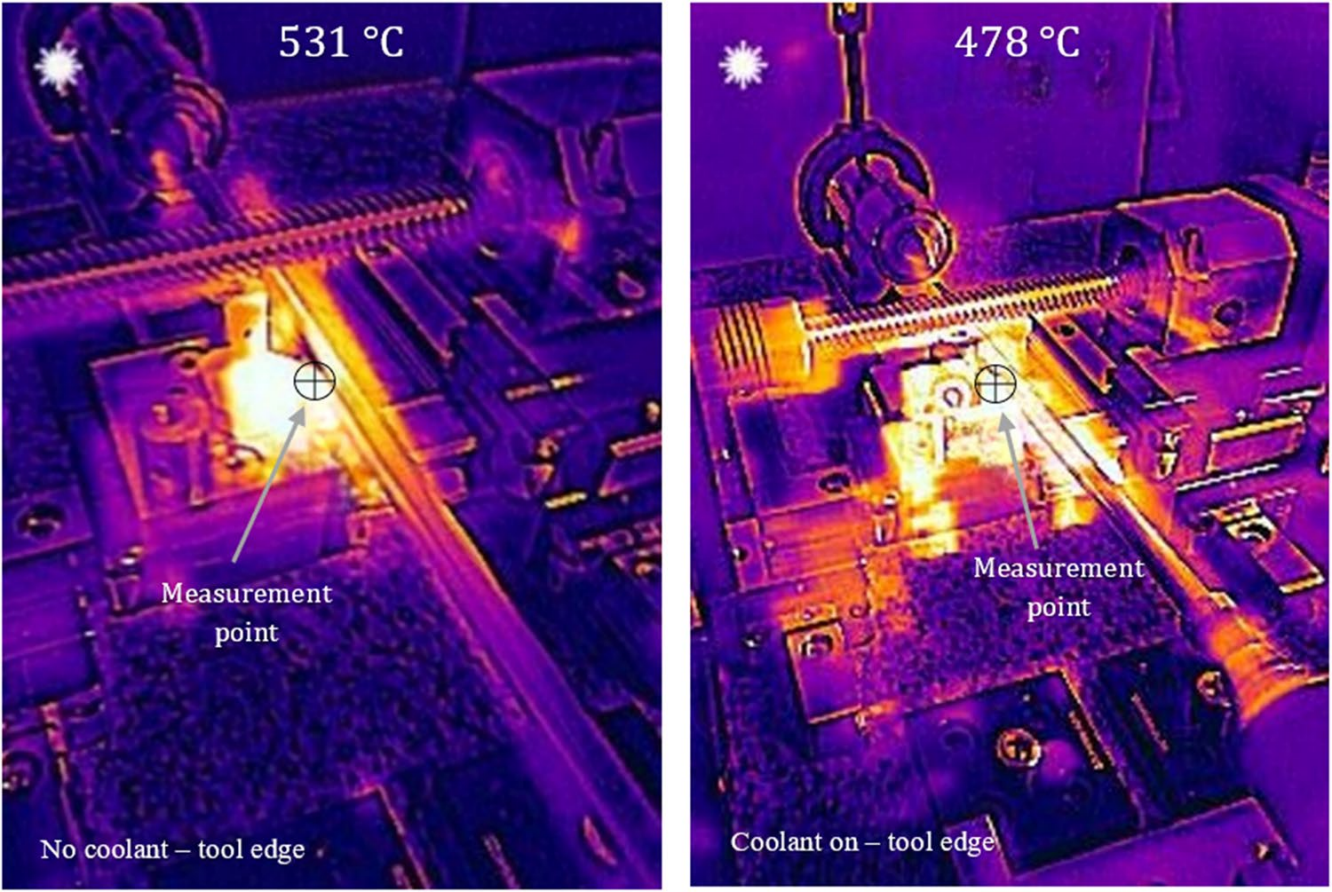
Thermographic images of the heat transfer for the tool edge at V_c_ = 900 m min.^−1^

Figure 15 shows the result of the cutting edge measurement. There is a clear variation in the temperature readings obtained with the cooling on (478 °C) and off (531 °C). The difference records a magnitude of 53 °C with V_c_ = 900 m min^−1^. This indicates that the MHD cooling system is working to transfer heat through the tool much more effectively than the tool itself. Thermal energy plumes are more prominent in the surrounding structure of the machine tool with the MHD active (Fig. 15). This can be accounted for through the larger heat energy dissipation through the local zone of the cutting tool system. The liquid gallium is carrying the generated thermal energy which is then radiated out into the immediate surroundings.

**Fig. 16:**
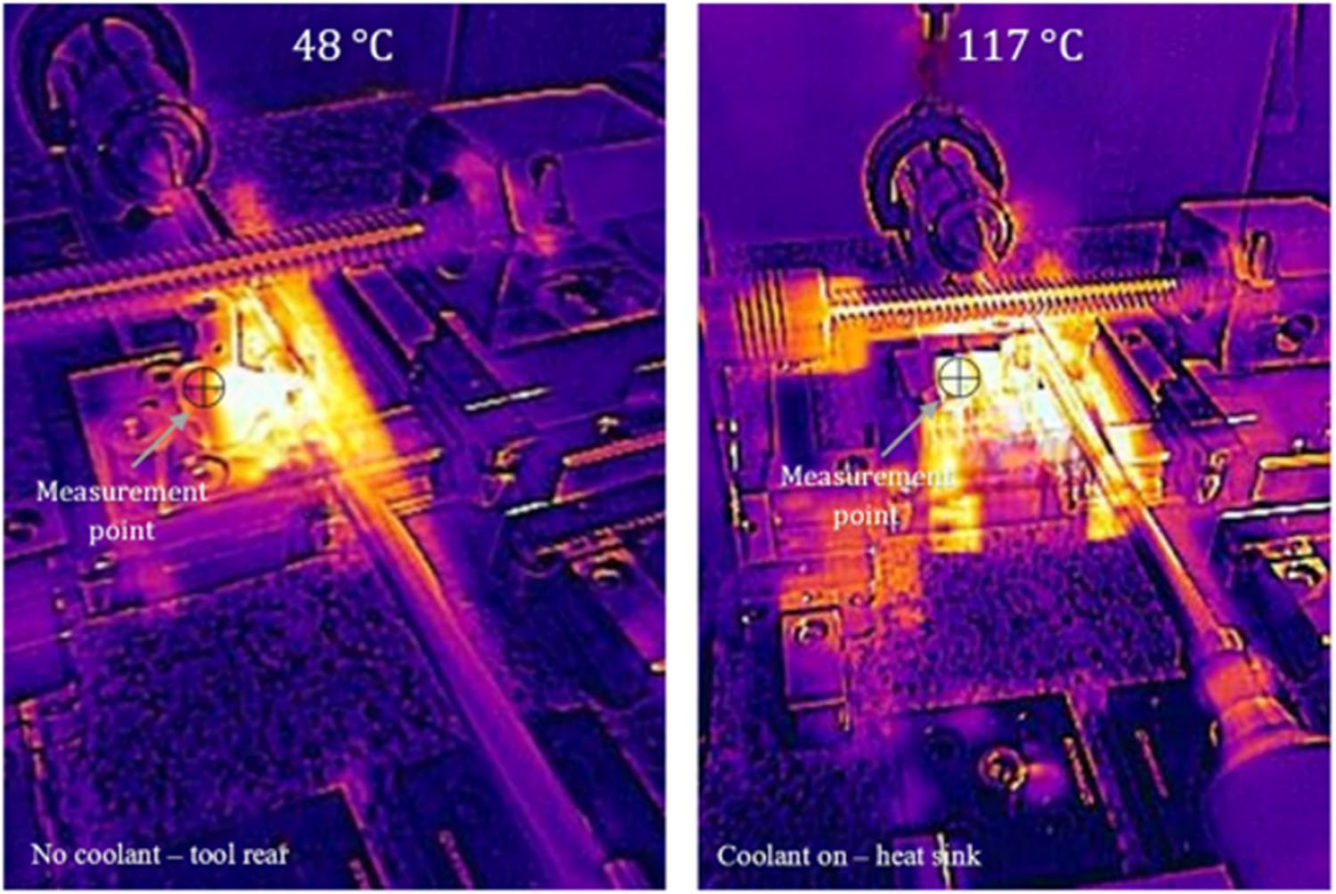
Thermographic images of the heat transfer for the heat sink at V_c_ = 900 m min^−^.^1^

As before, it was necessary to remove the aluminium housing unit to enable the target area to be measured with the thermographic imager. From Fig. 16, thermographic measurement of the cutting insert surface rear area without coolant is 48 °C. For the test with the MHD coolant on, the measurement obtained is 117 °C. This records a temperature difference of 69 °C. In this case, the magnitude of the heat plume. The reason for this observation is the superior thermal transfer capacity of the circulating liquid gallium, allowing for accumulated heat to be transported away from the cutting edge. This effect, combined with the hBN heat sink, allows for enhanced heat transfer via the solid and fluidic boundaries across the internal channel and through the heat sink which is transferred to the ambient air. This demonstrates that the MHD system can successfully remove heat via the internal liquid metal.

There is a general correlation between increasing the cutting speed and tool wear. It results in higher temperatures which results in faster tool wear, moreover, the cutting speed itself has the largest effect on the cutting temperature [41]. The interdependency of this relationship allows for thermal imaging of the cutting zone to provide data on the magnitude of heat generated. The method to measure the tool-workpiece interaction is positioning the thermal imager in such a way that it can record in situ, the dynamic cutting zone during the machining process [26].

### Fluidic comparison

**Fig. 17:**
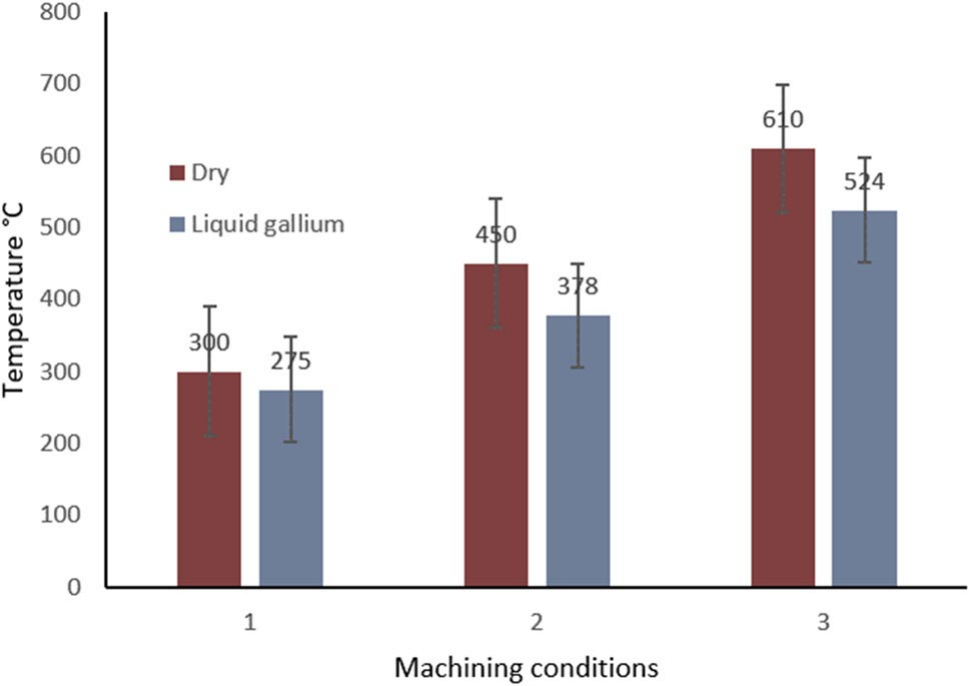
Temperature at the cutting edge for conditions of dry and cooling on, for the three different cutting speeds. V_c_ 1 = 250 m min^−1^, 2 = 500 m min^−1^, 3 = 900 m min^−^.^1^ [20]

In Fig. 17, the resultant data of the varying temperature profiles at the cutting edge of the insert is shown for cutting speeds *V*_*1*_, *V*_*2*_, *V*_*3*_. It can be seen that the liquid gallium reduced the local surface temperature, and this reduction in temperature increased as the cutting speed increased accordingly. This is likely due to the gallium being superior at heat transfer because of the increased velocity currents, and/or in combination with the thermal conductivity of the liquid gallium increasing with the rising temperature. The increase in heat does affect the liquid gallium thermal properties in respect to conductivity. Thus, this might suggest a correlation between the data obtained at higher cutting speeds. Further analysis might provide deeper insight to this thermal transfer phenomena, which may lead to engineering thermal transfer conditions within the cutting tool structure.

### Tool wear

A correlation exists between cutting speed and localised temperature which shows an exponential increase in metals [41]. However, in the case of ceramics this is not observed. The reason for this is that ceramic materials require high temperatures to plastically deform the uncut chip in order to effectively remove the layer. The higher cutting speeds favour the microstructural properties and thermomechanical behaviour of Al_2_O_3_. Notwithstanding this, ceramics like metallic cutting tools, experience wear when exposed to high temperatures and strains.

The creation of the crater pattern depends on the secondary deformation characteristics of the chip [41], which occurs when high frictional forces exist between the tool and chip, thus increasing localised heat. The active coolant serves to dissipate the high thermal energy and thus reduces the large frictional forces which accounts for the difference in wear patterns on the rake face. Welding of hot chips can occur along the rake face of the tool, this type of action was predominantly observed in dry cutting.

**Fig. 18:**
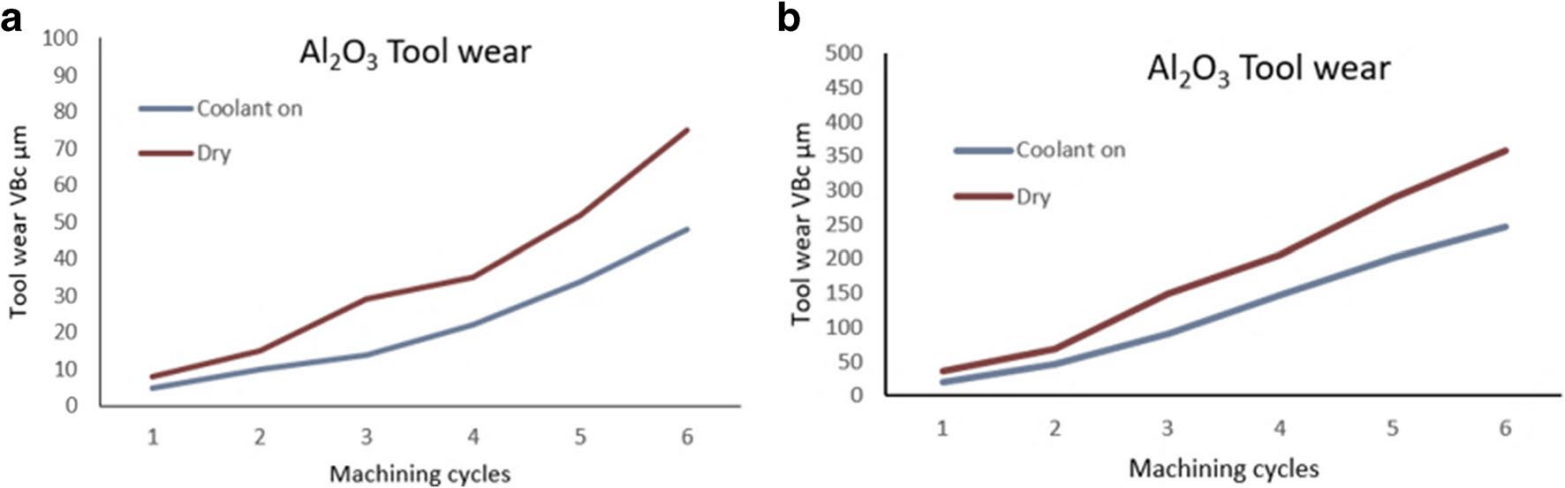
Corner wear VB_c_ rates under conditions of dry and internal cooling (**a**) Number of machining cycles for the cutting insert at V_c_ = 250 m min^−1^, and (**b**) at V_c_ = 900 m main^−^.^1^ [20]

**Fig. 19:**
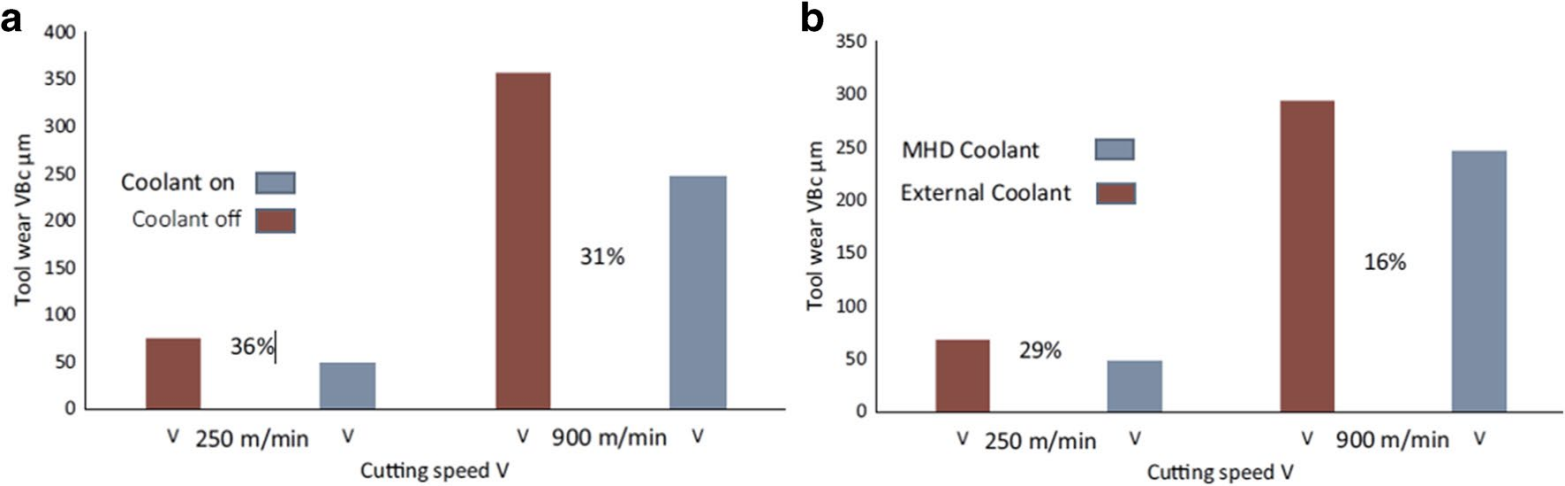
Tool wear reduction. (**a**) MHD cooling system vs no cooling. (**b**) MHD cooling system vs external cooling with liquid water [20]

For the first test, the corner wear rate VB_c_, corresponding to six cycles with V_c_ = 250 m min^−1^, recorded 75 µm with the coolant off and 48 µm with the MHD coolant on (Fig. 18). This represents a decrease of 36% in relative tool wear. As the cutting speed was increased to V_c_ = 900 m min^−1^, the corner wear rate VB_c_, obtained a value of 357 µm with the coolant off and 246 µm with the MHD coolant on. This represents a decrease of 31% in relative tool wear (Fig. 19a). These results indicate that the MHD coolant is actively transferring the heat away from the tool edge, and that the MHD cooling system provides for a measured increase in tool longevity relative to the ceramic tool under dry conditions without internal cooling.

In the second test, a liquid water-based coolant was applied to the cutting zone using a motorized pump which fed water at 2.5 mL s^−1^ at 20 °C (Fig. 19b). The cutting speeds are exactly as used in the previous conditions, namely, V_c_ = 250 m min^−1^, and V_c_ = 900 m min^−1^. When the external coolant was active, at V_c_ = 250 m min^−1^, the corner wear VB_c_ rate observed was 68 μm. When the MHD coolant is active, the resultant measurement gave 48 μm. This represents a decrease of 29% in tool wear difference. Increasing the cutting speed to V_c_ = 900 m min^−1^, the external coolant produced a corner wear VB_c_ rate of 294 μm, and with the MHD activated the resultant wear magnitude was 246 μm. The difference between the tool wear rate reduction with the MHD coolant relative to the external coolant being 16%.

### 316L workpiece

**Fig. 20:**
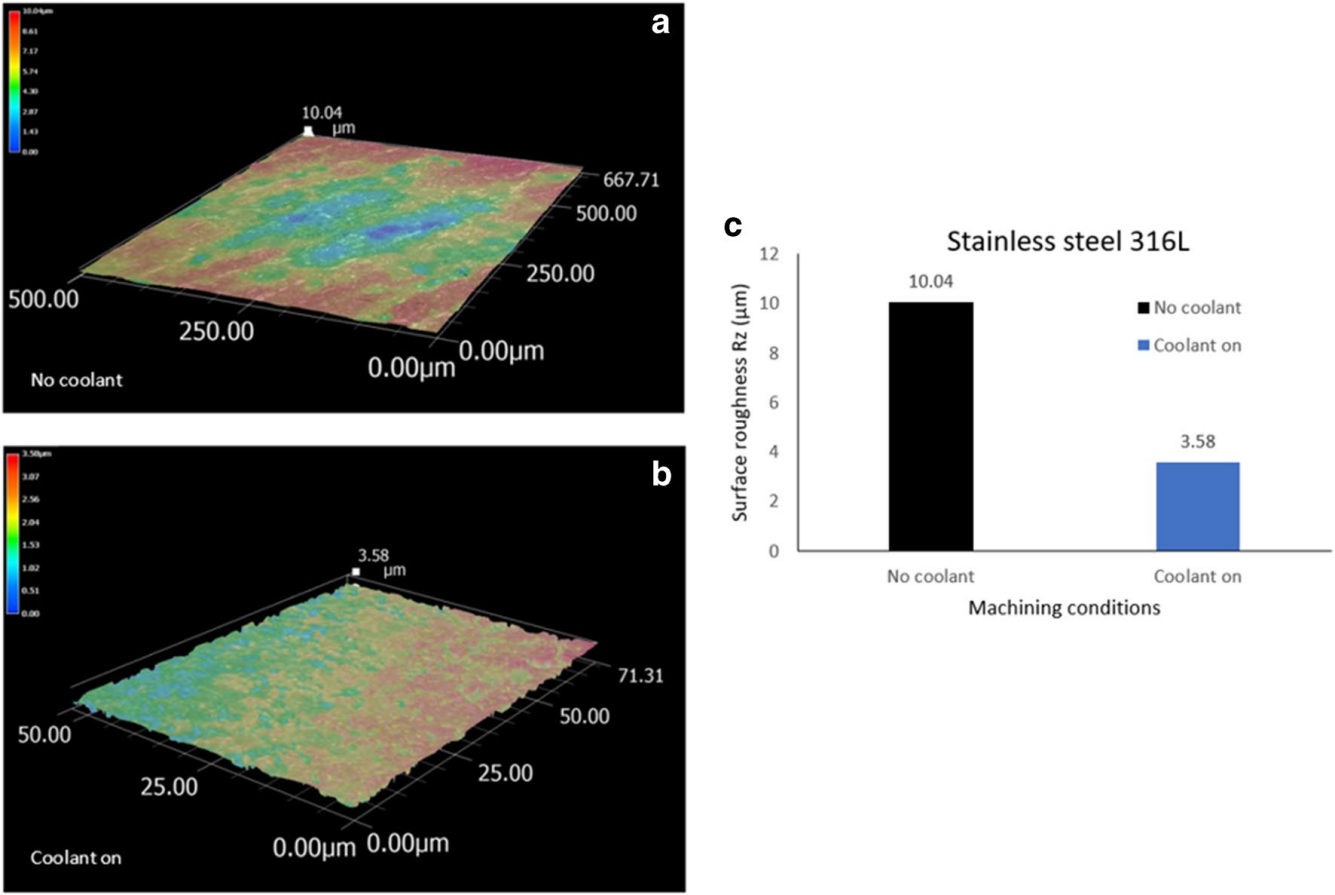
Topological surface measurements in R_z_ of the machined 316L alloy with (**a**) no cooling and (**b**) with MHD cooling system on at V_c_ = 900 m min^−1^. (**c**) Comparison of the 316L surface topologies under the conditions of no coolant and active MHD cooling system for V_c_ = 900 m min^−^.^1^

The two sets of tests showed varying differences in respect to the cutting speed insofar as the surface roughness of the workpiece reduced as the speed increased. This was expected as the ceramic tool operates better at higher cutting speeds. The first set of tests used the Al_2_O_3_ insert under dry machining conditions without the MHD cooling system on. The DOC and feed rate were kept constant as previously indicated, with three variants on the cutting speed magnitude as before (Table 3). The resultant surface roughness of the 316L workpiece with the coolant off is shown in Fig. 20a. The surface roughness at V_c_ = 900 m min^−1^ in Rz is 10.04. The second set of tests used the Al_2_O_3_ insert with the MHD cooling system on (Fig. 20b). As before, the same machining conditions are applied. The resultant surface roughness of the 316L workpiece at V_c_ = 900 m min^−1^ in Rz is 3.58. In terms of surface roughness comparison, Fig. 20c shows the difference between the two sets of tests. It can be seen at V_c_ = 900 m min^−1^, the difference in surface profile gives a magnitude of 6.46 in Rz between the two cases.

From these results the following conclusions can be drawn: for the case of lower cutting speeds there is marginal difference between the two machining tests. However, a roughly linear relationship develops corresponding to increased roughness in the case of dry machining compared to the inserts with the MHD cooling on. This makes sense as the higher cutting temperatures affect the tool-workpiece interactions. As the MHD cooling system removes the heat from the cutting edge more effectively, it reduces thermal ablation and the negative effects of the high cutting zone temperatures thus improving the cutting mechanism.

## Discussion

### Cutting speed, heat generation and wear in ceramic inserts

Attempting to implement a reduced rate of material removal using ceramic inserts constitutes one of the primary factors in both rapid tool degradation and workpiece damage [35]. This approach produces insufficient heat, and the result is a low rate of heat transfer in front of the tool. Workpiece annealing does not occur, resulting in high cutting forces and ultimately premature damage to the tool structure. Higher surface speeds with moderate feed rates and low DOC’s, allows for the generation of high heat in the cutting zone, and subsequent propagation of the thermal energy into the uncut material workpiece. This creates a plasticisation of the region in front of the cutting tool thus easing material removal. From a design perspective, because of the high strain on the ceramic insert, a stronger geometric configuration is preferred with high-speed machining in this case*.* However, because ceramics are brittle, they are also prone to chipping and microfractures which are exacerbated by excess thermal stress. The solution to this is a moderate heat transfer mechanism (thus avoiding thermal shock) using internal coolants. In particular, with austenitic 316L, the low thermal conductivity means the heat is not transferred into the chips efficiently. This results in an overheating of the cutting tool edge. Hence, the dry machining approach using ceramics requires a holistic approach to controlling the heat transfer process which in this case is in the form of an internal coolant.

For the case of Al_2_O_3_, the presence of water-based lubricants can impact wear rates, whereby tribochemical and sliding wear dominates, which for the latter is related to dissolution of the Al_2_O_3_ microstructure under contact points [18]. Polycrystalline oxide ceramics, which Al_2_O_3_ is a class of, typically have excellent wear resistance over metal inserts but this is dependent on the machining conditions and the workpiece material. Rapid transitions when machining the workpiece can result in rapid wear which was initially gradual. Furthermore, the length of time for this transition in wear to occur increases with smaller grain size and reduced loading [18, 35]. There is a gradual increase in internal stresses from increasing subsurface damage resulting from the tool-workpiece interaction, with eventual grain boundary fracture and grain pull-out produces fracture like wear patterning.

In terms of wear rates, smaller grain sizes in the Al_2_O_3_ microstructure are associated with lower abrasive wear rates [35]. For larger grain sizes, microfracture and fracture pull-out are evidenced producing rougher surface features. With smaller grain sizes (which was the case for the fabricated inserts), polishing of the surface was seen, indicative of plastic deformation during the wear process.

### The internal channel and heat transfer

**Fig. 21:**
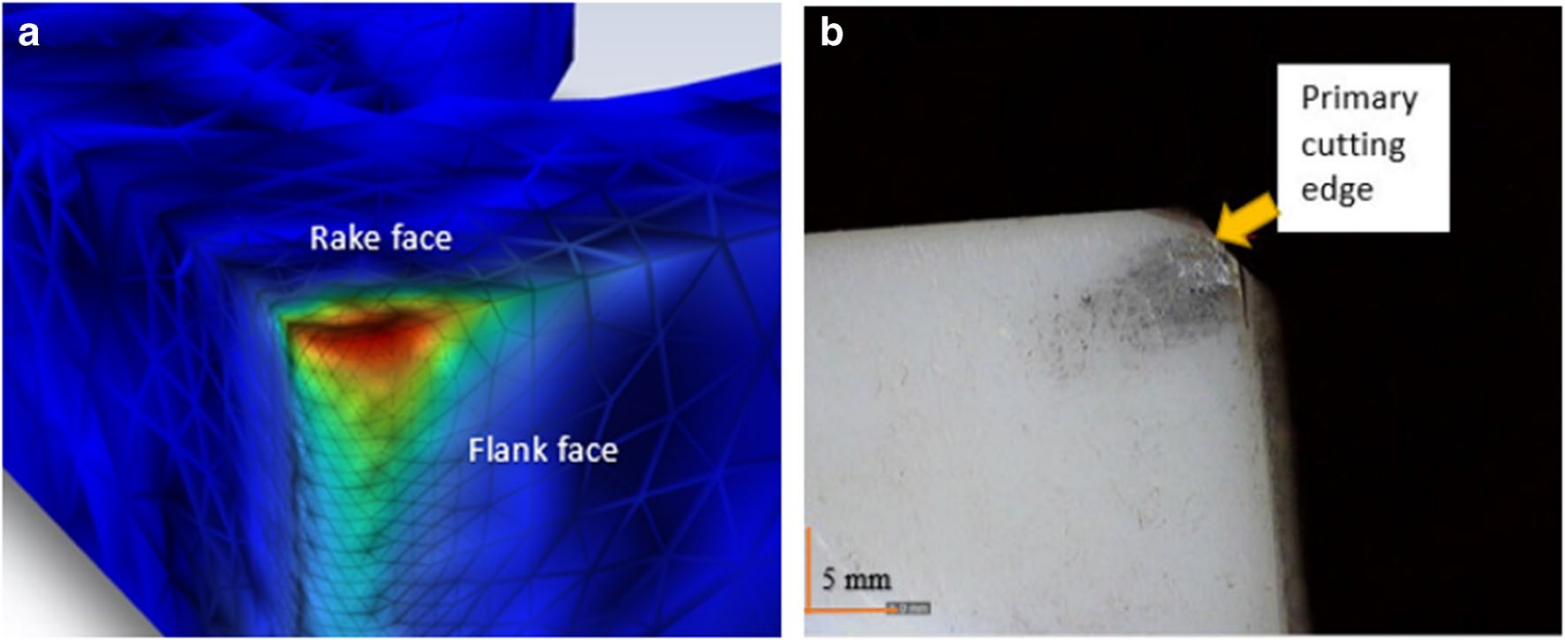
Heat impact on the cutting tool. (**a**) CHT model showing the location of the thermal energy at the region of the cooling channel surface. (**b**) Prototype Al_2_O_3_ insert exhibiting flank and rake wear profiles

To maintain structural integrity when designing the insert, the upper limit on internal channel distance from the flank was restricted to 0.7 mm and from the rake face 0.85 mm. The simulation model was constructed to allow for the maximum permissible distance the internal channel could be placed from the tool edge. However, this tool is a prototype, as such, any changes that subject the tool and indeed the entire machine configuration to variable forces may result in different results. The study used a novel MHD method to remove the thermal energy from the tool-chip interface. The liquid gallium velocity profile responds in accordance with the local conditions in the cutting zone. For example, an increase in cutting speed will invariably increase the local temperature, and in turn, the circulating liquid will experience an input of thermal energy which then produces an increase in the fluidic velocity within the chamber. This has the effect of removing the heat more efficiently through the turbulent convection currents circulating inside the insert. An example of the heat transfer effect in the CHT model and the prototype insert is shown in Fig. 21a and b respectively.

**Fig. 22:**
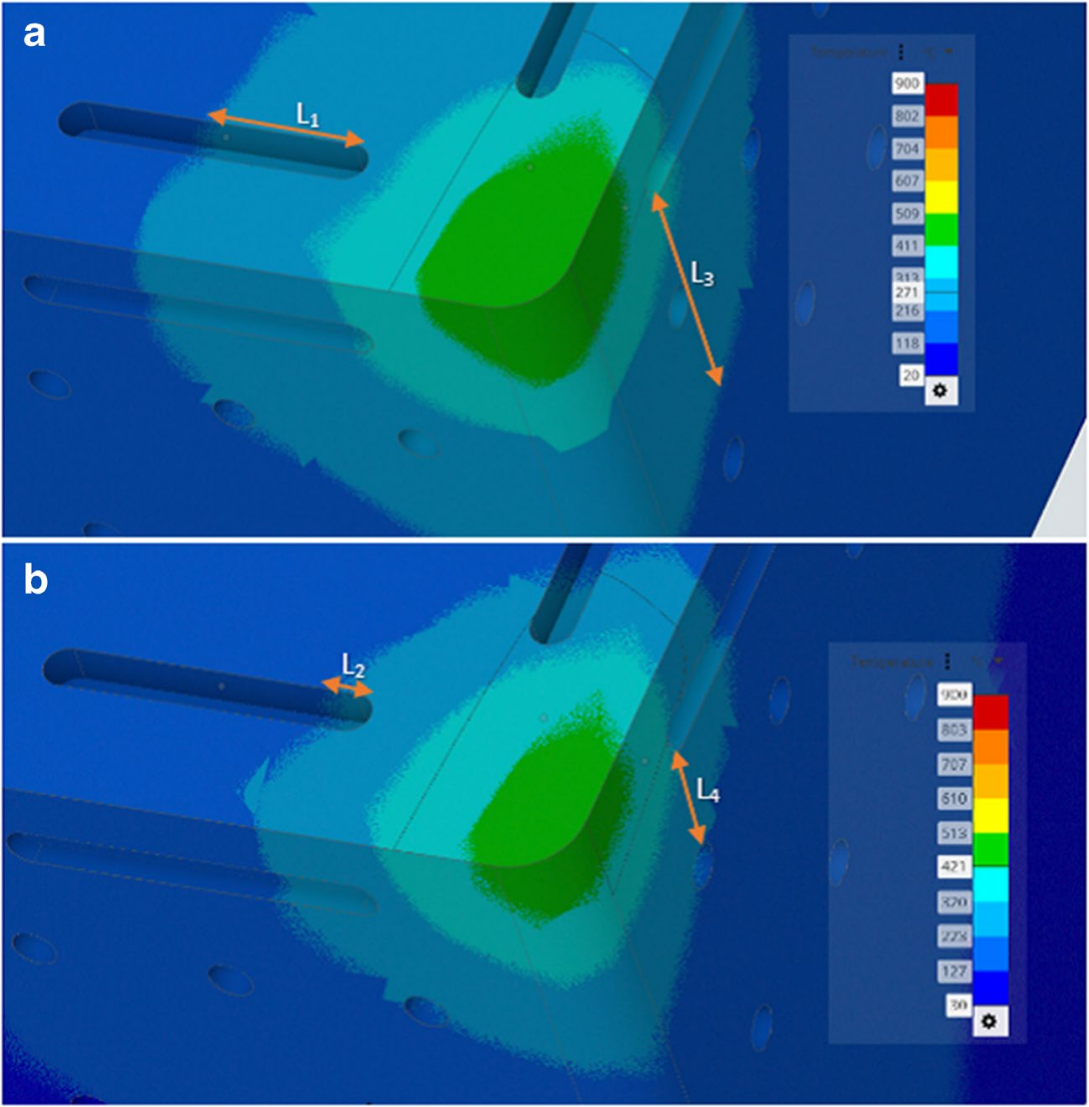
CHT simulation surface temperatures for (**a**) liquid water and (**b**) liquid gallium of the Al_2_O_3_ insert at an applied temperature of 600 °C

Comparative analysis of the temperature distribution contour across the tool surface for liquid water and liquid gallium under the same applied heating conditions. Figure 22a shows the temperature contour across the cutting tool surface edge for liquid water. The region of highest regional temperature is 489 °C. In Fig. 22b, the temperature contour for liquid gallium is lower as indicated by the reduced temperature under the same conditions at 436 °C. This indicates a surface temperature difference at an applied temperature of 600 °C (edge) of 53 °C. There appears to be a correlation between the increasing surface temperature and the rate of heat transfer for both fluids. This relationship may indicates that liquid gallium performs better at heat transfer when subjected to higher temperatures within the cutting zone relative to the liquid water based coolant under the same conditions. This is significant as it means the liquid gallium is more effective at removing thermal energy from the internal channel at higher temperatures.

In Fig. 22a and b, there are linear arrows located on the rake and major flank regions of the cutting insert edge. The rake face regions are represented by L1 and L2 for the liquid water and liquid gallium respectively. It is clear that the surface heat magnitude for L1 is larger than that of L2. Also, the major flank represented by the arrows L3 and L4 for the liquid water and liquid gallium respectively displays differing lengths which indicate a difference in surface temperature between the two fluids. Although this is a graphical comparison, the results can be visualised better when examined using the two sets of data plotted for the same region using the data obtained for the heat transfer efficiency of the two liquids.

### Correlation between temperature and tool wear

The optimisation of suitable cutting parameters, the cutting time and tool wear rates are important considerations in establishing an accurate measurements of surface roughness [26]. However, this study is primarily concerned with the magnitude of heat transfer that occurs using the MHD cooling system. Therefore, it is not within the scope of this work to include machining variables which can modify the resultant rate of tool wear or surface roughness. To do that would be prohibitively time consuming and add detailed complexity to identifying what is effectively a heat transfer problem under controlled conditions. In light of this, the cutting time, cutting parameters (including depth of cut and feed rate) are kept constant, apart from the cutting speed as previously stated. Also, for each measurement, one cycle of 100 mm cutting distance was used.

**Fig. 23:**
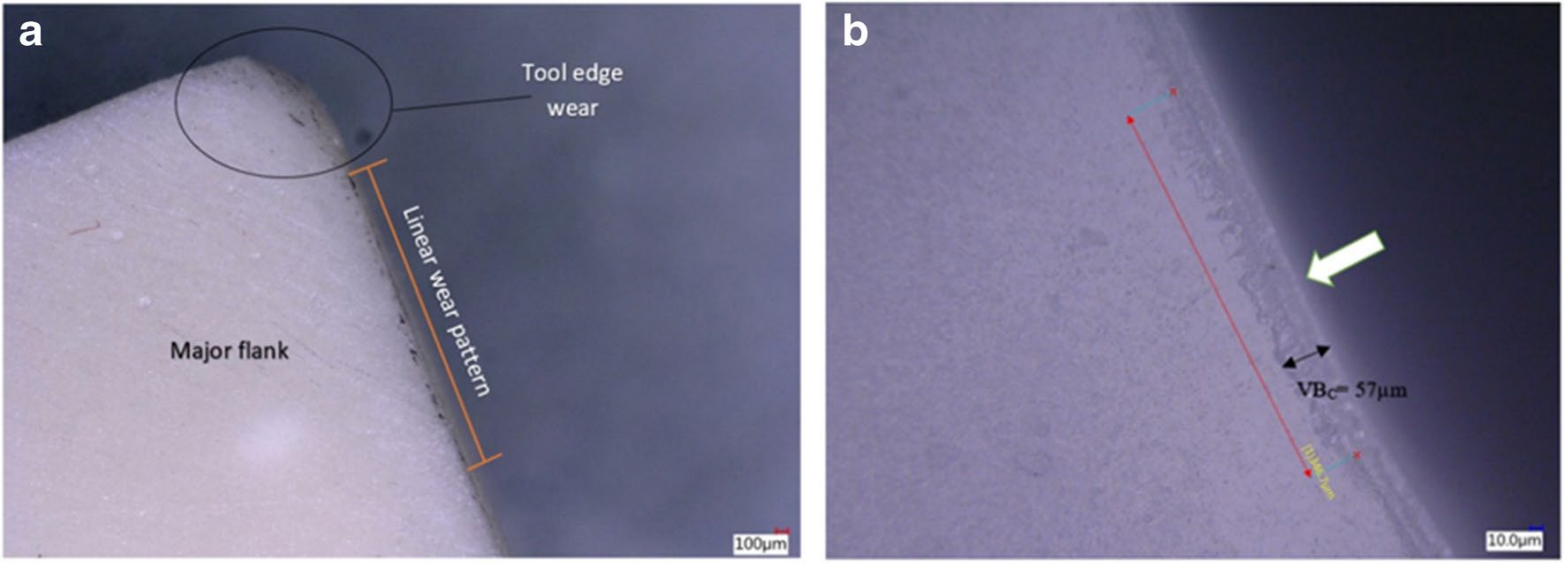
Images of the tool wear profile with MHD cooling system on for 10 cycles (**a**). The Al_2_O_3_ insert showing a region of adhesive wear on the major flank without cooling for 10 cycles (**b**)

The degree of tool wear directly impacts the surface roughness of the workpiece, in fact, the signature of the tool is transferred into the surface of the workpiece. So, as tool wear progresses, then in turn, the surface integrity is reduced [26]. One of the main causes of excessive tool wear is large amounts of thermal energy which occurs in uninterrupted cutting such as turning operations. It can be inferred that the higher thermal loading, which results primarily from the higher cutting speeds has a significant effect on the rate, type and location of tool wear (Fig. 23a,b). The higher wear rates caused by thermal ablation and frictional conditions has a cyclic effect; as the tool wear increases, so does the local temperature in the cutting zone. This in turn accelerates the tool wear until it becomes no longer possible to perform satisfactory material removal. Therefore, tool wear and temperature have a deep connection, mutually affecting each other.

### Temperature variation in the simulation model

Temperature contours revealed a drop in temperature as the heat travels away from the tool tip. When the cooling was activated the temperature distribution initially showed a similar thermal profile but after ~ 10 s, the temperature dispersed more rapidly across the surface of the tool. Fluid dynamics of the thermal transfer can be seen through the simulation of the liquid flow internally. When the heat reaches the liquid gallium, convection currents increase which carry the thermal energy away from the primary heat source further into the channel. Velocity vectors indicate that the rise in thermal energy increases the local fluidic motion removing the heat faster. As time increases, the global velocity reaches a lower equilibrium concentration, and this then provides a conduit for the heat to travel. At this point, the circulating liquid gallium is carrying the thermal currents toward the heat sink. The higher viscosity of the liquid gallium also contributes to maintaining the superior heat transfer rate when compared to liquid water.

### Heat transfer analysis

It should be noted that the geometry of the surface can affect temperature readings. Additionally, surface reflectivity is high within the cutting zone producing higher emissivity [41]. Consideration of this was included by taking the average of six readings over different locations. From this follows the requirement of accurate surface emissivity. Because the range of the material varies (metal-ceramic) it is difficult to apply the correct emissivity magnitude and collect the right temperature reading. Also, true emissivity is a dynamic phenomenon which depends on the material movement during the heat cycle [41]. Because of the camera location, the actual maximum temperature at the tool contact relative to the cutting edge is difficult to obtain and only one side of the cutting zone is visible at this point. Therefore, the reading may be affected. However, the aim of this study is to assess the heat transfer which necessitates the heat to be followed through the entire tool-heat sink system. Therefore, this was the primary approach employed in the study to reflect the mechanism of the MHD system heat transfer.

Thermal imaging of the tool-workpiece interaction showed differences in terms of the way thermal energy is dissipated. For the conditions of dry machining without the coolant system active, it reflects the poor thermal conductivity of the Al_2_O_3_ insert. In this case, the heat can only move through conductive means and raises the localised temperature of the tool-toolholder unit. As the cutting tool is fed along the surface of the 316L workpiece, the high speed and frictional conditions produce increasing wear on the surface of the insert. Contrast with the MHD cooling active. There is a clear change in the spatial location of the heat energy. The tool workpiece shows a lower heat transfer value which indicates the heat is being removed through dual conductive-convective system. The liquid gallium acts to transfer the heat from the tool through the internal channel via convection currents.

Interestingly, it was also found that increasing the cutting speed resulted in a corresponding increase in heat transfer. This suggests that the MHD cooling unit is more effective as the convective currents circulate. This was also observed in the simulation studies.

**Fig. 24:**
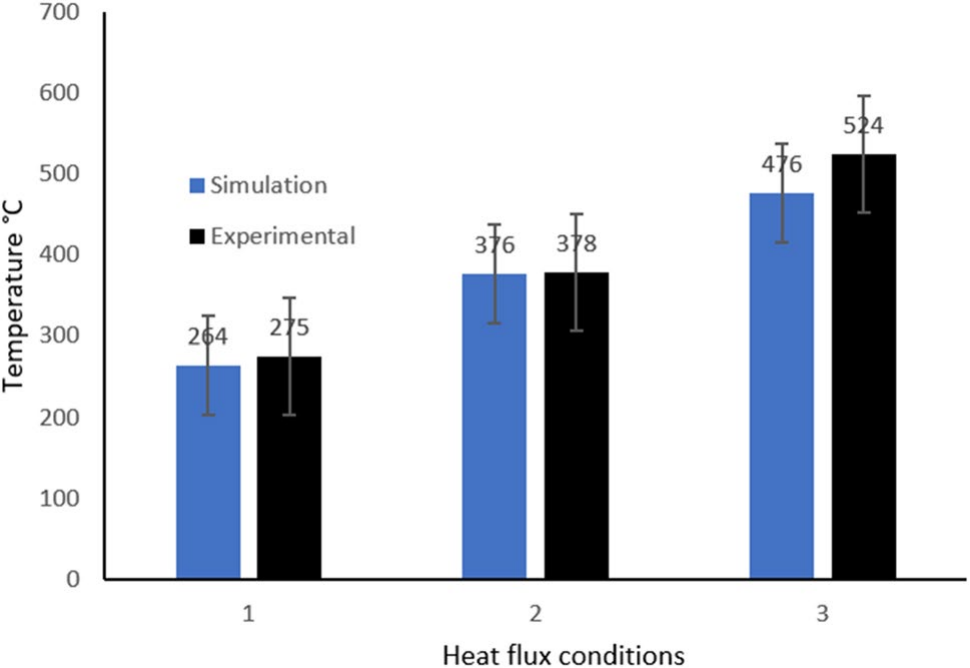
Comparison of simulated versus experimental heat measurements at the primary cutting edge for liquid gallium, under three heat flux conditions (V1, V2, V3)

In Fig. 24, a comparison of simulated and experimental data obtained for liquid gallium is presented. There is some deviation between the two sets of data for all three heat flux conditions. It cannot be definitively accounted for, but there is likely an oversimplification of the simulation parameters and conditions used in the model. It also is likely that some, if not all the measurements taken during the experimental tests were flawed in some way. This is due to the inherent difficulty in accurately measuring the target zone of the cutting insert in-situ during the machining process. Aside from the dynamic elements of the machine-tool interface during the cutting action, there is also the challenge of targeting the correct area of the tool surface region itself. Therefore, it remains unclear as to the exact magnitude of heat transfer variance there exists between the simulated model and the physical prototype test.

The results are similar in all cases, with the largest difference observed when the cutting speed was V_3_ = 900 m min^−1^. The other two cutting speeds, V_1_ = 250 m min^−1^ and V_2_ = 500 m min^−1^, showed closer comparison. However, this does not mean that the experimental tests validate the CHT data without consideration. Firstly, the difficulty in taking measurements in situ of the dynamic machining process can easily lead to erroneous results as mentioned. The targeting of the laser at a moving object is inherently challenging. Furthermore, it is difficult to factor in the degree of error associated with the measurement. Another problem is the positioning of the camera relative to the cutting insert. Numerous angles were tested and the most promising location (the one used to take the final measurement) had itself problems during the capturing of the image. Therefore, at first sight, the data appears to support quite well the simulated results, caution should be used in assigning a definitive correlation between the two sets of tests. The largest variant in temperature values is at *V*_*3*_ = 900 m min^−1^. The reason may be the effects of the increased heat produced in the cutting zone which potentially influenced the thermal imaging, insofar as radiative heat combined with the dynamic motion of the cutting tool contributed to the actual measurement.

### Suggestions for further developments

#### Patterning on the tool surface

Research by [10, 42] has indicated a positive effect in tool wear reduction with the use of.

**Fig. 25:**
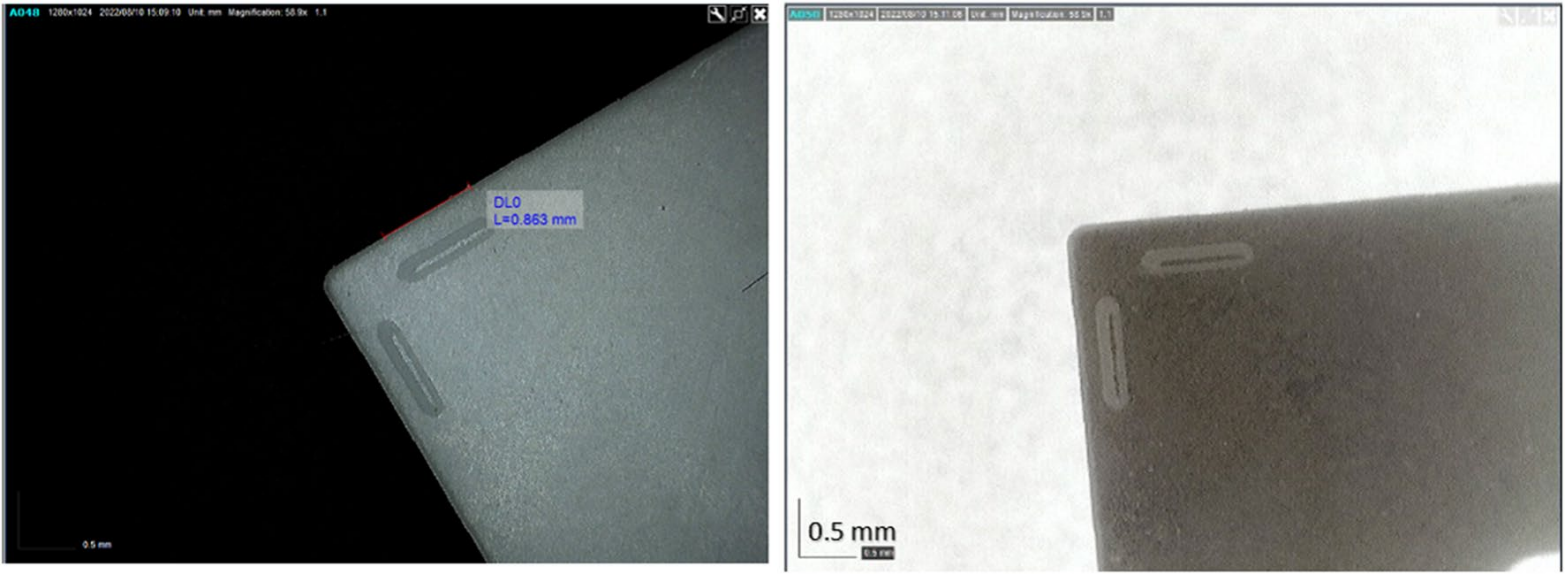
Optical and monochromatic images of the insert rake face with linear patterning

MQL in combination with textured patterns. For this study, linear patterns were fabricated into the flank and rake faces of the cutting tool (Fig. 25). This was done for the purpose of testing the potential of the additive manufacturing process on its effectiveness in forming surface features. However, the patterning can also as act a mechanism to reduce surface wear on the cutting tool in conjunction with a hexagonal boron nitride lubricant, which has excellent friction reduction capabilities. It would be instructive if further studies could examine how the combined MHD system with surface patterning plus MQL behaved under the same conditions. It would be possible to use both as an enhanced mechanism in thermal management of tool wear.

#### ***Enhanced heat transfer in the Al***_***2***_***O***_***3***_*** insert***

The MHD system transferred thermal energy via the circulating liquid gallium, however, the ultimate effectiveness of the design is restricted within the boundaries of the inserts’ maximum heat conductivity. Therefore, to enhance the cooling system for practical machining applications, it is necessary to integrate into the structure a mechanism to increase the localised heat conductivity and by extension improve the heat removal rate.

**Fig. 26:**
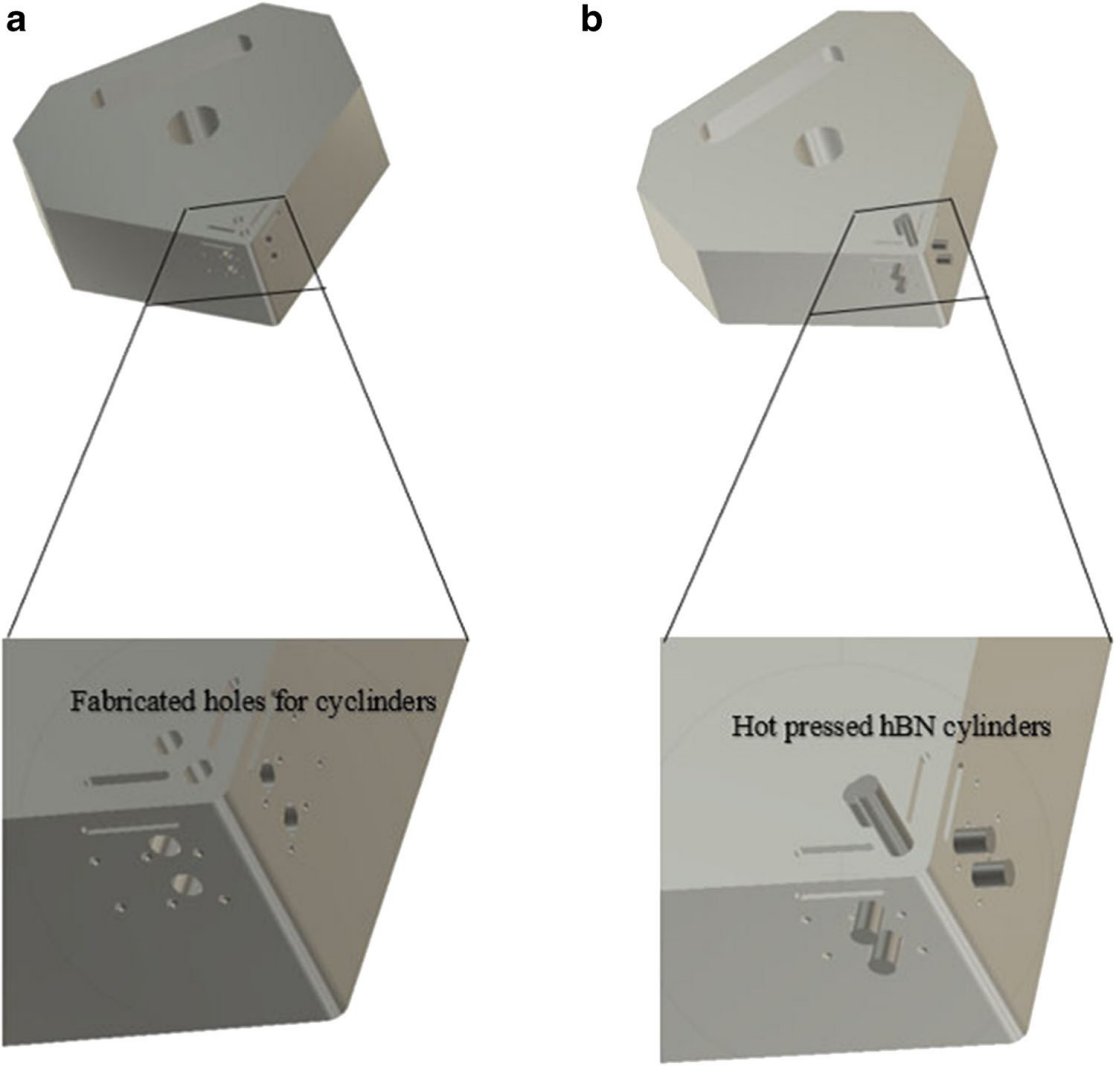
CAD model showing the proposed method of enhanced heat transfer. (**a**) Location of the holes, (**b**) insertion of the hBN cylinders

The system under consideration has the following material properties (Table 8). The compatible materials that can be implemented in conjunction with liquid gallium is restricted. By judiciously selecting the optimum material that can provide the desired material properties and cost effectiveness, hexagonal boron nitride was chosen as a suitable material for this application. In this application, it is necessary that the heat conduit material has a solid structure that is stable, heat resistant and exhibits excellent thermal conductivity. Because the Al2O3 cutting tool will be exposed to high temperatures (300–600 °C) within the cutting zone, the powdered form of hBN would not be useful in this case, as the high heat would cause the particles to descend into the internal chamber and render the heat conductivity unchanged. To address this issue, hot pressed hBN could be formed into cylindrical parts which are then inserted into the fabricated holes in the Al2O3 body (Fig. 26).

**Table 8:** Relevant properties of hot pressed hBN

hBN Properties	Magnitude
Density	2.0–3.0 g/cm^3^
Operating temperature	900–2000 °C
Thermal conductivity	30–80 W/mk

hBN is chemically stable in high heat conditions with the atomic structure represented by a hexagonal lattice with covalent B-N bonds. The hBN layers are held together by Van der Waals forces, which are relatively weak resulting in anisotropic properties, as such, this provides for the superior transfer of heat. It is also an excellent lubricant (~ 0.3 coefficient of friction) and exhibits good thermal shock resistance.

The limitations of the aluminium oxide thermal conductivity requires further research through implementation of the suggested hot pressed hexagonal boron nitride cylinders, or through alternative means to improve the heat transfer within the microstructure. The MHD cooling system provided a heat path through the hBN heat sink which convectively exchanged thermal energy with the ambient air. However, more effective heat removal methods are necessary to enhance this mechanism. In particular, thermal equilibrium would eventually be reached due to the limiting heat sink capacity. Therefore, a better heat exchanger is recommended to improve the MHD internal cooling design with the liquid gallium coolant.

## Conclusions

Experimental tests were performed using liquid gallium in a prototype cutting tool under controlled conditions on a custom-made turning machine. The tests were conducted using a novel magnetohydrodynamic cooling system on stainless steel 316L and then compared against the same insert under dry machining and external cooling conditions. The study clearly indicated that liquid gallium outperforms as a heat transfer agent, and by extension reduces tool wear, in all cutting speed variations. Moreover, it was found that as the cutting speed increases, the heat increases, and the effectiveness of the heat transfer rate also increases accordingly. This would suggest that at higher temperatures, the magnetohydrodynamic cooling system removes thermal energy more efficiently. Further studies are required to better understand the underlying mechanism of heat transfer in this type of system.

The numerical models developed along with the experimental tests results support the hypothesis that liquid gallium can transfer heat through an internal cooling mechanism in ceramic inserts. The conclusions of this study can be drawn as follows.

1. Liquid gallium provides a means to transfer thermal energy at a more effective rate compared to the liquid water-based coolant using the MHD cooling system embedded within an aluminium oxide cutting insert, which is supported by the numerical model.
2. The numerical modelling results showed that liquid gallium outperformed liquid water in terms of heat transfer over three distinct temperature ranges.
3. Experimental tests validate the simulation results with some degree of variation, which is likely attributed to a combination of modelling parameters and the method of measuring the cutting zone temperature using the thermographic imager.
4. Under dry machining conditions, with V_c_ = 250 m min^−1^, the corner wear VB_c_ rate observed was 75 µm with the coolant off, and 48 µm with the internal MHD coolant on. This represents a decrease of 36% in tool wear. When the cutting speed was increased to V_c_ = 900 m min^−1^, the corner wear VB_c_ rate showed 357 µm with the coolant off, and 246 µm with the MHD coolant on. This represents a decrease of 31% in tool wear.
5. When external cooling using a liquid water-based coolant was added, the results showed at V_c_ = 250 m min^−1^, the difference between the tool wear rate reduction with the internal coolant relative to the external coolant was 29%. When the cutting speed was increased to V_c_ = 900 m min^−1^, the difference observed between the internal liquid gallium coolant relative to the external coolant was 16%.

Liquid gallium is an effective cooling agent for use in internal cooling cutting tools as a sustainable solution to thermal management in subtractive machining. Limitations on its use, despite excellent heat transfer potential relates to compatibility of the substrate material. This was addressed through the aluminium oxide ceramic which compliments the materials used and the manufacturing method employed. Other challenges identified the material properties of aluminium oxide, where its relatively low thermal conductivity combined with the form of hexagonal boron nitrite heat sink limits the effectiveness of the cooling system over time. Nonetheless, both sets of results show that as a heat transfer mechanism for use as a form of sustainable manufacturing, liquid gallium combined with a magnetohydrodynamic drive has potential for applications in thermal management in cutting tool technology.

## Figures and Tables

**Fig. 27:**
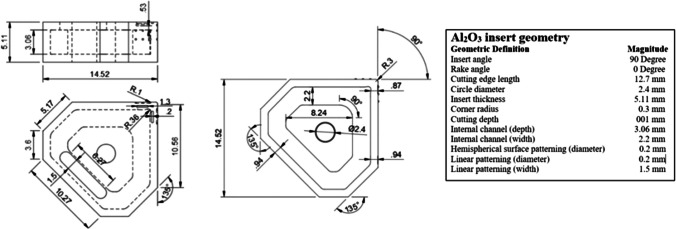
The geometrical specifications of the cutting insert
